# A three process approach for insomnia-like awakenings

**DOI:** 10.1371/journal.pone.0360640

**Published:** 2026-11-04

**Authors:** Joe Rowland Adams, Maia Angelova

**Affiliations:** Sir Peter Rigby Digital Futures Institute, Aston University, Birmingham, United Kingdom; Mae Fah Luang University School of Anti Aging and Regenerative Medicine, THAILAND

## Abstract

Mathematical modelling of sleep is a growing field, with an increasing number of applications to sleep disorders. However, despite its high prevalence and under-diagnosis worldwide, insomnia has not been among these disorders. We chart the development of sleep models and investigate the hypothesis that they can be simply modified to model insomnia, ultimately introducing a new model focusing on nocturnal awakenings as a starting point of insomnia-like behaviour, based on a three process approach. We find that several of these models can make promising steps towards producing nighttime awakenings characteristic of many types of insomnia with minor modifications, but that none can fully reproduce every key criterion with only minor adaptations. In particular, we find that the stability of the sleeping state is an important factor in more complex contemporary neuronal population models’ abilities to incorporate awakenings, with both permanently stable and permanently non-stable sleep proving to be obstacles. Instead, we base our new model on the simpler foundational two process model, incorporating an original, quantitative wakefulness drive. We further analyse the sensitivity of its new parameters and the statistical properties of the nighttime awakening phenomena it produces. This new drive includes both physiology-based arousal impulses and environmental and psychological disturbances, and provides the foundation of a new approach to future quantitative modelling of insomnia phenotypes.

## 1 Introduction

Sleep is a complex biological state characterised by behavioural, physiological and electrophysiological parameters. Good quality sleep is a vital component of general physical and mental health [1–3], and fuller understanding of sleep disorders, both in terms of causes and treatments, would be of significant benefit to both sufferers and healthcare organisations. Mathematical models of the processes underlying the phenomenon of sleep and its related disorders could be a vital tool for advancing this understanding.

Mathematical models of sleep have been under development for over 40 years (see for example [3–7]), successfully reproducing many of sleep’s dynamical features and representing many different physiological interpretations of the processes involved, and have been applied to understand multiple sleep disorders. In addition to mathematical models, data-driven models have been widely utilised in this field, which are not governed by phenomenological or other rules but by the patterns arising from the experimental data.

Despite this success, there has been little-to-no progress towards a model of insomnia symptoms, and there is much still to be understood about the phenomenon of sleep in general. In this paper we will review the development of key contemporary mathematical models of sleep, present results exploring their dynamical features, investigate whether simple modifications drawing from these analyses to these models can reflect core insomnia behaviours, and ultimately introduce a new three process model of sleep affected by awakenings.

To understand the gap between contemporary sleep models and a model of insomnia, we must first discuss in more detail the physiology of the processes of sleep and insomnia. Key mechanisms involved in sleep and transitions between sleep and wakefulness include:

- Circadian regulation. Circadian rhythms in activity are apparent in all animals – diurnal species such as humans, zebrafish and flies primarily sleep at night, while nocturnal species, such as mice, primarily sleep during the day. The circadian clock can be synchronised to the environment by different cues, or zeitgebers [8], such as light, temperature, stress and exercise, food availability, and social interactions. However, it also persists in the absence of the environmental time cues – demonstrating the existence of an intrinsic regulatory mechanism. This (dis)regulation alone is linked to numerous sleep disorders, including insomnia, e.g., due to a circadian phase shift [9].
- Homeostatic regulation. When wakefulness is extended, sleep pressure, or the urge to sleep, accumulates and only dissipates during subsequent sleep.
- Multiple sleep stages. These include REM (rapid eye movement) and substages of non-REM, which alternate during sleep. REM sleep is thought to play a role in brain development, memory and processing, and in general lasts for a minority of a sleeping period in comparison to non-REM stages.
- Orexin/hypocretin (Hcrt), a neuropeptide primarily produced in the lateral hypothalamus, which plays a crucial role in the regulation of REM sleep and appetite. By inhibiting REM sleep and, to a lesser extent, promoting wakefulness, orexin helps maintain a balance between sleep and wakefulness [10].
- Motivational regulation. When motivated, humans can stay awake and engage in various cognitive and physical activities, such as reading a book, cooking, working on a grant, far beyond their physiological bedtime while ignoring homeostatic and circadian sleep drives [11].

Physiological causes of insomnia have not been definitively identified, but there have been many hypotheses of the possible causes. These have ranged from genetic and other physiological factors to psychological and environmental stressors to disruptions of sleep-regulatory mechanisms and neurobiological mechanisms. All of these factors may contribute to a predisposition for an individual to experience insomnia and as exacerbating factors that may trigger or worsen any insomnia episodes [2,12], with clinical research suggesting that the circadian drive, homeostatic drive, neuronal functions, behaviour and environment all play a significant role in the experience of insomnia [13]. It is additionally commonly comorbid with other sleep disorders, such as obstructive sleep apnoea [14]. This has significant implications for both experimental analysis and simulations, as it impairs our ability to observe and represent ‘pure’ insomnia. In this paper we aim to reproduce symptoms clinically associated with such ‘pure’ insomnia, but a consideration of the impact of these co-morbidities remains an important future consideration. Despite the uncertainty as to the exact cause of the condition, its formal description is given in reference to three symptoms [15,16]:

- Difficulty initiating sleep.
- Difficulty maintaining sleep.
- Early morning awakenings.

Insomnia is a highly variable disorder, featuring many different phenotypes and co-morbidities with other disorders [17]. However, with so much still unknown about the physiological causes, and even processes, of insomnia, subjective reporting plays a large role in insomnia diagnosis. It is in large part the result of these complexities and uncertainties that little progress has been made towards a mathematical model. We therefore focus primarily on the phenomena of involuntary nocturnal awakening, an important aspect of insomnia but not in of itself totally descriptive, as a starting point for the necessary shift from models focused on typical sleep to sleep featuring disturbances common to insomnia.

Thus, the aim of this paper is to develop a mathematical model describing involuntary nocturnal awakenings, as a first step towards improving our understanding of insomnia more widely. To identify effective mechanisms to accomplish this we will review the development of major contributions to mathematical modelling of sleep and investigate the hypothesis that only minor modifications to a selection of these models are required for them to be able to describe involuntary nocturnal awakenings, finally introducing such a model based on these investigations. In Section 2 we will review the development of important contemporary sleep models. In Section 3 we discuss what successful awakening models of insomnia may look like, present original results on perturbative additions to existing sleep models for this purpose. Finally, we introduce an original model of sleep disturbed by involuntary nocturnal awakenings founded on the conclusions of these investigations, conduct a sensitivity analysis of this model, and compare summary statistics of its phenomenological outcomes to insomnia. The Supporting Information additionally includes S1 File, expanding on the analysis of the existing sleep models’ stability in Section 2, and S1 File, expanding on the sensitivity analysis of the original model also presented in Section 3.

## 2 Review of sleep modelling

We sleep following the change of light to dark in cyclic patterns, known as sleep-wake cycles. These cyclic patterns motivated the development of mathematical models. These have included the two process model for sleep timing, based on the interactions between a homeostatic sleep drive and the circadian rhythm [18,19], coupled oscillator models for the same interactions [20,21], the reciprocal interactions model for REM sleep cycling [22,23] and models that investigate the dynamics of sleep-wake regulatory networks inspired by computational neuroscience modelling neuronal population activities [5,24,25]. We will now review the most significant of these contributions to the modelling of insomnia.

### 2.1 The two process model

The first major development in phenomenological mathematical models we will discuss is the two process model [18,19]. The two process model considers sleep as the product of two processes: a homeostatic drive that builds during waking periods and falls during sleep, which encourages a transition to sleep at high levels and wake at low levels, and a circadian drive, which promotes the need to sleep during the night and suppresses it during the day. This formalism of two interacting biological clocks, of a building and decreasing sleep pressure modified by a circadian rhythm, came to dominate sleep models, and continues to form the basis of most contemporary models. Its contemporary relevance was recently further highlighted in [26], where its significant mathematical similarity to more recent network physiology models was demonstrated.

This model may be mathematically formulated as the two processes


$$\begin{array}{cc} \frac{dS}{dt} & =-\frac{1}{\chi_{w}}S+\frac{\nu }{\chi_{w}}\text{ during wake,} \end{array}$$ (1)

$$\begin{array}{cc} \frac{dS}{dt} & =-\frac{1}{\chi_{s}}S\text{ during sleep,} \end{array}$$ (2)

$$\begin{array}{cc} C(t) & =\cos\omega t, \end{array}$$ (3)

where *S* is the homeostatic drive, $\chi_{w}$ and $\chi_{s}$ are decay constants of *H* during wake and sleep periods respectively, ν an equilibrium constant of *H* during wake periods, *C* the circadian drive and ω the frequency of that drive. Switching between the sleeping and waking states is governed by the thresholds


$$\begin{array}{cc} S^{+}(t) & =S_{0}^{+}+aC(t)\text{ switches to sleep,} \end{array}$$ (4)

$$\begin{array}{cc} S^{-}(t) & =S_{0}^{-}+aC(t)\text{ switches to wake,} \end{array}$$ (5)

where $S_{0}^{+}>S_{0}^{-}$ are the threshold constants, and *a* is a constant governing the influence of the circadian drive.

This model is of course a significant simplification of the true physiological processes of sleep in favour of a simple encapsulation of key phenomenological features, in particular realistically timed transitions between sleep and wake. Indeed the authors themselves have suggested several important avenues of future development for this approach, including differences in the evolution of the processes in different brain areas, feedback interactions between the two processes, e.g., mediated by the suprachiasmatic nucleus, and inclusion of the role of metabolism in determining these processes [6].

One recent development based in the two process model attempted to extend it to include REM and non-REM sleep cycles [27]. This used a nested doll model of exponential growth and decay functions, at one scale governing the macroscopic sleep/wake processes, and nested within that governing REM/non-REM sleep types. The macroscopic process is defined as


$$\begin{array}{cc} S(t) & =S_{u}-(S_{u}-S_{b})\exp\left(-\frac{t-t1}{Tb}\right)\text{ during wake,} \end{array}$$ (6)

$$\begin{array}{cc} S(t) & =S_{l}-(S_{d}-S_{l})\exp\left(-\frac{t-t2}{Td}\right)\text{ during sleep.} \end{array}$$ (7)

The parameters $S_{u}$ and $S_{l}$ define the upper and lower asymptotes of the macroscopic waking process respectively, $S_{b}$ and $S_{d}$ the lowest value of the decay and highest value of the growth respectively and *Tb* and *Td* the phase constants of the exponential growth and decay respectively. The microscopic processes meanwhile are defined as


$$\begin{array}{cc} s(t) & =s_{u}-(s_{u}-s_{b})\exp\left(-\frac{t-t1}{tb}\right)\text{ during NREM,} \end{array}$$ (8)

$$\begin{array}{cc} s(t) & =s_{l}-(s_{d}-s_{l})\exp\left(-\frac{t-t2}{td}\right)\text{ during REM,} \end{array}$$ (9)

where the meaning of the lowercase parameters correspond to those for the uppercase macroscopic processes.

Another direction for two process-inspired models is to add further processes or inputs, most often motivated by other physiological functions. This has included core body temperature [28], core body temperature and light input [29], sleep inertia (a period of reduced alertness after waking) [30,31] and environmental inputs in general [32]. Another recently introduced example is the two mode three drive (2m3d) descriptive paradigm [33]. In this paradigm both sleeping and waking states are separated into two different modes, m0 and m1, representing neurodynamic-metabolic processes associated with either dissipating or reinforcing homeostasis. This is in addition to three drives that govern the sleep/wake transitions. The first is the homeostatic drive, as in the original two process model, which grows while the system is in m1 and triggers a transition to m0 once the drive reaches a sufficient value, during which mode the drive decreases. The second is the chronophasic drive, which incorporates both the circadian drive already accounted for in the two process model and ultradian drives that result in, e.g., the REM and non-REM sleep phases, modulating sleep propensity according to the time of day. The final is the most original contribution – the afferent drive. This drive incorporates all other influences not accounted for in the others, from external sensory input to the subject’s emotional state, to simulate temporary significant disturbances to sleep propensity.

It is this addition of a third drive to a broadly two process model formalism that could offer significant greater capacity of such models to replicate a wide range of sleeping states and disorders. While it is not clear what the precise cause of insomnia, and many other sleep disorders, is, short-term perturbations such as the afferent drive in the 2m3d model may have the flexibility and capability to generate the inability to initiate or maintain sleep, or even maintain a waking state, that characterise insomnia and many other sleep disorders.

### 2.2 The flip-flop model

The flip-flop model focuses on state transitions between sleeping and waking and different sleep phases [8,34,35]. The flip-flop model posits that mutually inhibitory interactions of wake-promoting and sleep-promoting neurons produce a state similar to a flip-flop switch in an electrical circuit [10]. This concept has been used as a helpful framework to explain interactions between wake- and sleep-promoting circuits that minimise intermediate states between sleep and wakefulness. However, intermediate and dissociated arousal states do exist, for example in narcolepsy and REM behaviour disorder [11].

It is based in the premise that there are sleep- and wake-promoting neurons, each of which are localised in different areas of the brain and are mutually inhibitory. Hence, wake-promoting activity builds until reaching a transition-triggering threshold, suppressing sleep-promoting activity as it does, and vice versa. This produces a flip-flopping transition between the sleeping and waking states, as suggested by the name of the model. The principal wake-promoting neuronal populations identified in this framework are the cholinergic, monoaminergic and glutamatergic neurons, with significant influence from the orexinergic neurons. The ventrolateral and median preoptic neurons meanwhile are mostly responsible for generating sleep activity.

This model was used to theorise that sleep disorders may result from damage to the different areas of the brain containing these different neuronal clusters. For example that narcolepsy could result from impairment of the neurons responsible for stabilising the current state, causing excessive flip-flopping, or that impairment of the sleep-promoting neurons may be a cause of insomnia [36].

This framework for understanding wake and sleep, in particular the dynamics of the transitions between these states, has been a defining contribution to the field since its introduction. Rempe et al. developed a quantitative mathematical model for sleep based in the flip-flop model [37]. This model utilises Morris-Lecar system of equations [38] to calculate the activity of five cell populations based on those highlighted in the original flip-flop model, each with a contributory role to determining the sleep/wake state, and five drives.

There have also been many developments to include more complex dynamics and recent findings on the roles of additional neuronal groups. For instance, hypothalamic circuitry was found to have a role in tuning the waking and sleeping periods according to their specific conditions [39]. When a waking period is particularly intense for example, this circuitry acts to produce a similarly intense period of sleep following this to compensate. This mechanism was hence referred to as a switch with a spring, encapsulating the fact that intensity of the character of the sleep response is partially determined by the pressure built up in the preceding wake period.

It has also extended beyond just considering the transition from sleep to wake to transitions within sleep, such as REM-non-REM sleep transitions. In this case, the switching is similarly caused by the relative activity of different neuron populations in the brainstem, some associated with REM and others with non-REM [40]. Both non-REM and REM activity are promoted by GABAergic neurons from different parts of the brainstem. As in the case of the wake-sleep switch, these populations are mutually inhibitory, giving rise to the flip-flop behaviour. The former is additionally influenced by the noradrenergic, serotoninergic and orexinergic neurons, and the latter by ventrolateral preoptic and cholinergic neurons.

### 2.3 Physiology-inspired network models

The phenomenological contributions discussed in the previous subsections were vital contributions to the study of sleep, establishing frameworks through which the overall observable dynamics of the system could be understood. This has led to a wide variety of approaches being applied to do so, including wave models [41], machine learning models [42], data-driven models, such as the Unified Model of Performance and state classifications [43–54], agent models [55], coupled oscillator models [56–58], integrate-and-fire models [59], stochastic models [60–62] and neuronal population models [24,25,36,63–79]. We will now discuss some of these approaches in more detail, in particular the neuronal population approach, which has become the dominant methodology in the quantitative modelling of sleep.

#### 2.3.1 Neuronal population network models.

The next major development in sleep models we consider was a move to neurophysiology-inspired modelling [63–65]. This approach adopts the fundamental framework of the two process model, and indeed has been shown to be essentially identical to it in the slow timescale regime of sleep-wake transitions [80]. It was also directly derived from and inspired by the identification of flip-flop neurophysiology, and therefore is designed to mathematically generate the flip-flop behaviour described in the previous section.

This approach treats neural clusters as the models’ subjects, the relative activities of which are responsible for transitions between different sleeping and waking states, but additionally develops differential equations for the evolution of the mean soma voltages of these neuronal populations. In these models, two neuronal masses were considered, the monoaminergic (MA) group, associated with wake-promoting influences, and the ventrolateral preoptic nucleus (VLPO), associated with sleep-promoting influences, with additional interactions with the acetylcholine related (ACh) group. In addition to these two neuronal masses, the model also included the influence of a homeostatic drive and circadian drive, building on the two process model. The voltage of the MA group, $V_{m}$, and VLPO group, $V_{v}$, were formulated as


$$\begin{array}{cc} \tau_{m}\frac{dV_{m}}{dt}+V_{m} & =\nu_{mv}Q_{v}+\nu_{ma}Q_{a}, \end{array}$$ (10)

$$\begin{array}{cc} \tau_{v}\frac{dV_{v}}{dt}+V_{v} & =\nu_{vm}Q_{m}+\nu_{vc}C+\nu_{vh}H. \end{array}$$ (11)

Here, $\tau_{j}$ are the decay times of the respective neuronal groups *j*, $\nu_{jk}$ the weights of the input from group *k* to *j*, where subscript *a* denotes the ACh group, $Q_{j}$ the mean firing rate, *C* the circadian drive and *H* the homeostatic drive. The mean firing rates are defined as


$$\begin{array}{c} Q_{j}=\frac{Q_{\text{max}}}{1+\exp\left(-\frac{V_{j}(t)-\theta }{\sigma^{\prime }}\right)}, \end{array}$$ (12)

where $V_{a}$ is constant, θ is the mean firing threshold and $\sigma^{\prime }\pi \sqrt{3}$ is its standard deviation. The homeostatic drive is


$$\begin{array}{c} \chi \frac{dH}{dt}+H=\mu Q_{m}, \end{array}$$ (13)

where χ is the characteristic time for somnogen clearance and μ is a constant. Finally, the circadian drive is C=sin(ωt), where ω is the frequency.

Through these equations, the model reproduces the dynamical transitions from sleep to wake and vice versa, but was not designed to incorporate further phenomena such as REM and non-REM phases. The key evolution of these models from the two process model is the continuous dynamic nature of the sleep/wake state switching. In the two process model the transition is triggered by process S reaching a hardcoded threshold. In these models the inhibitory/excitatory interactions between the neuronal populations lead to slow evolutions of the voltages of the sleep- and wake-associated groups, eventually reaching a tipping point in which there is a rapid transition from sleep/waking. The sleep state is thus defined as when the voltage of the sleep-associated group (here the VLPO) is greater than the wake-associated (here the MA), and vice versa for the waking state.

Finally, we introduce a further development of this model. This further development, presented in Fulcher et al. [36], is at the forefront of the application of this flip-flop model consisting of networks of neuronal populations. As such, it will be one of the models analysed further in Section 3. This further development included an orexinergic neuronal group to regulate the sleep/wake transitions, represented as a new population with voltage $V_{x}$, as also identified in the flip-flop model [36]. This resulted in modified neuronal group voltage equations of


$$\begin{array}{cc} \tau_{m}\frac{dV_{m}}{dt}+V_{m} & =\nu_{mv}Q_{v}+A_{m}+\nu_{mx}Q_{x}+\xi_{m}(t), \end{array}$$ (14)

$$\begin{array}{cc} \tau_{v}\frac{dV_{v}}{dt}+V_{v} & =\nu_{vm}Q_{m}+A_{v}+\nu_{vc}C+\nu_{vh}H+\xi_{v}(t), \end{array}$$ (15)

$$\begin{array}{cc} \tau_{x}\frac{dV_{x}}{dt}+V_{x} & =\nu_{xv}Q_{v}+A_{x}+\nu_{xc}C. \end{array}$$ (16)

In addition to adding a third neuronal group, further influences not directly included in the model, including the ACh group, are generalised into the constants $A_{j}$, and noise terms $\xi_{j}(t)$ are added to the original two groups. As can be seen by comparison to Equations 10–11, the changes to the mathematical structure of the model are limited to these aforementioned inclusions, and the influence of the new orexinergic group on the MA population. It is this new influence that helps to stabilise the existing model in such a way as to produce more realistic sleep/wake transitions, exhibiting the same continuous dynamical flip-flop switch as in the original models, but with the added richness of dynamics of the third population.

#### 2.3.2 Diniz Behn and Booth model.

Thanks to its capacity to quantitatively predict the evolution of the sleeping and waking states, including any perturbations to the ordinary healthy state that may be introduced, while remaining physiologically grounded, this modelling approach has become a major part of the field and continued to be developed and applied to new contexts since its introduction.

The next development discussed here also took a physiologically-inspired networks of differential equations approach, incorporating even further physiological functions and shifting the focus from differential equations of voltages influenced by mean firing rates to differential equations of firing rates influenced by neurotransmitter concentrations [24].

Perhaps most significantly, this further model aimed to include REM and non-REM sleep states. In addition to the sleep-wake cycles governed by the circadian rhythms (24 hours), propagated by the brain’s suprachiasmatic nucleus (SCN), within a sleep episode, these additional rapid eye movement (REM) and non-REM (NREM) rhythms occur in the transitions between slow wave sleep with a period of approximately 90 minutes.

The neuronal groups considered to achieve this were the dorsal raphe (DR), locus coeruleus (LC) and the wake active (WR) subgroups of the laterodorsal tegmental nucleus and pedunculopontine tegmental nucleus (LDT/PPT), all of which are wake-promoting, the ventrolateral preoptic area (VLPO), which is sleep-promoting, and the REM active subgroup of the LDT/PPT. The firing rates of these populations were modelled through the equations


$$\begin{array}{cc} \frac{dF_{LC}}{dt} & =\frac{LC_{\text{max}}\gamma_{LC}\left(A,-N,-G\right)-F_{LC}}{\tau_{LC}}, \end{array}$$ (17)

$$\begin{array}{cc} \frac{dF_{DR}}{dt} & =\frac{DR_{\text{max}}\gamma_{DR}\left(A,-S,-G\right)-F_{DR}}{\tau_{DR}}, \end{array}$$ (18)

$$\begin{array}{cc} \frac{dF_{VLPO}}{dt} & =\frac{VLPO_{\text{max}}\gamma_{VLPO}\left(-N,-S,-G\right)-F_{VLPO}}{\tau_{VLPO}}, \end{array}$$ (19)

$$\begin{array}{cc} \frac{dF_{R}}{dt} & =\frac{R_{\text{max}}\gamma_{R}\left(A,-N,-S,-G\right)-F_{R}}{\tau_{R}}, \end{array}$$ (20)

$$\begin{array}{cc} \frac{dF_{WR}}{dt} & =\frac{WR_{\text{max}}\gamma_{WR}\left(A,-G\right)-F_{WR}}{\tau_{WR}}, \end{array}$$ (21)

where $\gamma_{X}(Y,-Z)=0.5\left(1+\tanh\left(\frac{g_{Y,X}C_{Y}-g_{Z,X}C_{Z}+\delta (t)-\beta_{X}}{\alpha_{X}}\right)\right)$. The parameters $g_{X,Y}$ weight the influence of the concentration $C_{X}$ on the firing rate $F_{Y}$, δ(t) is a noise term, $\beta_{X}$ and $\alpha_{X}$ are constants and *h* is the homeostatic drive, defined as


$$\begin{array}{c} \frac{dh}{dt}=H[F_{LC}+F_{DR}-\theta_{W}]\frac{1-h}{\tau_{hw}}-H[\theta_{W}-F_{LC}-F_{DR}]\frac{h}{\tau_{hs}}, \end{array}$$ (22)

where *H* is the Heaviside function, $\tau_{hw}$ and $\tau_{hs}$ are the growth and decay constants, respectively, and $\theta_{W}$ is the waking threshold. Finally, the concentrations are calculated as


$$\begin{array}{c} \frac{dC_{X}}{dt}=\frac{\tanh\left(\frac{F_{Y}}{\gamma_{X}}\right)-C_{X}}{\tau_{X}}, \end{array}$$ (23)

where $\gamma_{X}$ is a constant and $\tau_{X}$ is the time constant of neurotransmitter release. With the addition of these further populations, this model was able to simulate not just sleep/wake transitions, but also REM/NREM within the sleeping state. The mechanism of the sleep/wake, and now also REM/NREM, transitions functions as in [36] in the previous section, i.e., via a continuous dynamical flip-flop switch of relative neuronal firing rate magnitudes. It has additionally been applied to understand the variability in human sleeping patterns [68], an important consideration for the likewise highly variable symptoms of insomnia.

#### 2.3.3 Diniz Behn and Booth simplified network model.

The final development in this modelling approach discussed here is a simplified network model of the Diniz Behn and Booth model we discussed in the previous section, also making variable firing rates and concentrations its main constituent components, driven also by a homeostatic, but no circadian, drive, however in this case departing further from a physiological basis [25,67,81]. Rather than simulating the firing rates of several different neuronal groups and the concentrations of their corresponding neurotransmitters, this model consists of a single equation representing the cumulative firing rates of all neurons responsible for promoting wake (W), REM sleep (R) and non-REM (N) sleep. Hence while departing somewhat from precise physiological analogues, the model is a significant mathematical simplification of its predecessors. These equations are


$$\begin{array}{cc} \frac{dF_{W}}{dt} & =\frac{W_{\text{max}}\left(0.5\left(1+\tanh\left(\frac{g_{NW}C_{NX}+g_{RW}C_{RX}+\zeta (t)-\beta_{W}}{\alpha_{W}}\right)\right)\right)-F_{W}}{\tau_{W}}, \end{array}$$ (24)

$$\begin{array}{cc} \frac{dF_{R}}{dt} & =\frac{R_{\text{max}}\left(0.5\left(1+\tanh\left(\frac{g_{WR}C_{WX}+g_{NR}C_{NX}+g_{RR}C_{RX}+\zeta (t)-\beta_{R}}{\alpha_{R}}\right)\right)\right)-F_{R}}{\tau_{R}}, \end{array}$$ (25)

$$\begin{array}{cc} \frac{dF_{N}}{dt} & =\frac{N_{\text{max}}\left(0.5\left(1+\tanh\left(\frac{g_{WN}C_{WX}+\zeta (t)-\beta_{N}}{\alpha_{N}}\right)\right)\right)-F_{N}}{\tau_{N}}. \end{array}$$ (26)

The concentrations are determined by


$$\begin{array}{c} \frac{dC_{YX}}{dt}=\frac{\tanh\left(\frac{F_{Y}}{\tau_{YX}}\right)-C_{YX}}{\tau_{YX}}, \end{array}$$ (27)

and the homeostatic drive by


$$\begin{array}{c} \frac{dH}{dt}=\frac{H_{\text{max}}-H}{\tau_{hw}}H[F_{W}-\theta_{W}]-\frac{H}{\tau_{hs}}H[\theta_{W}-F_{W}]. \end{array}$$ (28)

## 3 Our approach to insomnia-like awakenings

In this section we will first discuss the challenges involved in the construction of mathematical models of insomnia. We then investigate the hypothesis that existing general sleep models may be induced to exhibit specific core symptoms consistent with acute and chronic insomnia only (without any co-morbid sleep disorders) with the introduction of external perturbations. These models, previously reviewed in Section 2, were not intended to produce insomnia-like phenomena. We will present original results to test this hypothesis, and possible further pathways to developing models of insomnia-like awakenings. Building on the conclusions of this investigation, we then introduce an original model of physiologically, environmentally and psychologically driven awakenings, based on the current understanding of insomnia.

First, we must address the question of what a successful model of insomnia may look like. One of the most commonly reported symptom of insomnia is of course waking from sleep during the night. These awakenings may be followed by a return to sleep relatively quickly, but may also last for a significant period. Therefore a successful model of insomnia must allow for both short and long timescale awakenings. Another, and related, common feature of insomnia is a difficulty initiating sleep, whether at the beginning of the night or following an awakening. Similarly, a difficulty waking is a common symptom. Therefore a successful model must also be able to incorporate unpredictable variation in the start/end of the sleeping period. Due to both its reported prevalence by insomnia sufferers and being the most significant departure from the ordinary functioning of sleep models, we will focus on generating awakenings lasting for long and short periods in the following models. This behaviour alone would be far from sufficient to fully describe insomnia, however we will focus on this as the primary hurdle in transition from a model of sleep, to a model of insomnia. We additionally hypothesise that if nighttime awakenings meeting the above criteria can be successfully generated, a similar method to that which produced it may be used to add variability in the sleep-wake transitions. The simplest external perturbation that may achieve this is an additional driving factor that promotes waking activity, activating at variable times for variable durations, which we will now examine in application to three of the previously reviewed models. While it is not the only method that may produce such phenomena, indeed perhaps not even being the most likely to do so, we will investigate this first as the simplest approach. Additionally, if this method were to prove unsuccessful, the nature of this may help to define the nature of the challenges to other, more complex approaches. This approach will therefore focus on generating symptoms common to maintenance insomnia, i.e., a difficulty maintaining sleep, but may have further relevance to other insomnia presentations, such as onset insomnia if applied at the beginning of a transition to sleep, rather than during a sleeping period.

The four models we will apply this approach to are Fulcher et al. [36], Diniz Behn and Booth [24], Diniz Behn and Booth simplified networks [25,67] and the two process model [18,82], respectively. Each of these external perturbations will be made such that they are as consistent as possible with the mathematical schema of the original model, while also balancing this with the goal to be as mathematically simple as possible.

These perturbations, even if successful in producing awakenings of variable length and timing, will not constitute a complete model of insomnia as a whole, or even necessarily of specific phenotypes of insomnia. They will however give an indication as to whether these approaches, common to contemporary sleep modelling, can form an appropriate basis for modelling insomnia in any of its phenotypes, and whether a much-hypothesised additional drive within this scheme could be sufficient to bridge the gap from sleep to insomnia models.

### 3.1 Inclusion of awakenings in sleep models

We first consider the Fulcher et al. network model [36]. Of the major sleep models we have discussed in the previous section, this was the first based in a shift further towards a physiological basis for its modelling components.

The most immediate and significant limitation of this model to a fuller understanding of insomnia is that this model does not include any different stages of sleep. Nevertheless, as one of the most advanced and notable of the physiologically-inspired network approaches, the model offers important insights into the process of sleep. In order to induce the most important feature of insomnia, short-timescale nighttime awakenings, we include a third drive directly influencing the firing rate of the wake-associated monoaminergic brainstem nuclei.

This is of the form


$$\begin{array}{c} \sigma_{1}(t)\Delta V_{m}+\sigma_{2}(t)\zeta_{m}(t) \end{array}$$ (29)

where $\Delta V_{m}$ is a constant, $\zeta_{m}(t)$ is noise sampled from a uniform distribution and $\sigma_{1}(t),\sigma_{2}(t)\in \{0,1\}$, added to Equation 14. The switch variable $\sigma_{1}(t)=1$ when an awakening is triggered and $V_{m}$ has not reached the level associated with waking, and 0 otherwise. Similarly $\sigma_{2}(t)=1$ when an awakening has been triggered, $V_{m}$ has reached the level associated with waking and has been at that level for less time than the desired length of the awakening, and 0 otherwise. Physiologically, this therefore represents a noisy stimulation of the monoaminergic neurons. This is a similar approach to that taken in [83], which applied perturbations to a predecessor model of the one we consider here to both the MA and VLPO populations. It is hence consistent with the underlying theory of the original model, in that the drive to wake is incorporated as an additional drive in the evolution of the MA population firing rate, mediated by a switch, just as the original model is itself derived from the flip-flop switch approach.

Fig 1 shows a single example nighttime awakening of approximately 30 min over a 48 hr period using this approach. This was produced using $\Delta V_{m}=0.01$ mV. This was found to be of significant magnitude to produce an awakening, i.e., for the MA neurons’ firing rate to overtake that of the VLPO neurons, while producing an MA neuron firing rate in the awakening period that is comparable in magnitude to during normal waking periods. The impact of a $\Delta V_{m}$ of different values was investigated in the range [0, 0.02] mV in intervals of 0.001 mV, with all other parameters, including the timing of the activation of the $\Delta V_{m}$ drive, kept constant. This investigation found that $\Delta V_{m}>0.007$ mV was required to produce an awakening, with weaker perturbations failing to bring an end to the sleep state, and stronger ones producing only marginally longer awakenings. This finding is summarised in Table 1. This awakening can hence be produced by a relatively simple perturbation to the existing model. Additionally, despite leading to an MA firing rate of 98 /s compared to 2.6 /s, the awakening produced by $\Delta V_{m}=0.02$ mV lasted only 0.1 s longer than the minimal awakening of $\Delta V_{m}=0.008$ mV, at 0.15 s and 0.05 s respectively. The beginning of an awakening is defined as the point the MA neurons’ firing rate exceeds the VLPO neurons’, and the end as the inverse case. This shows that the sleeping state of the model is highly stable, meaning that it is rapidly restored after the additional drive causing the awakening is switched off with no additional intervention. This is both desirable and undesirable for modelling insomnia; in some circumstances an awakening is followed by sleep, which is easily accomplished with this model, while in others it is not, which would be more challenging to replicate as a result of this stability.

**Table 1:** A summary of the investigation of perturbation parameters and the thresholds required to produce an awakening in the Fulcher et al., Diniz Behn and Booth and Diniz Behn and Booth simplified network models.

Model	Perturbation parameter	Investigated range	Critical threshold
Fulcher et al.	$\Delta V_{m}$	[0, 0.02] mV	>0.007 mV
Diniz Behn & Booth	$\Delta V_{m}$	[0, 20000] /s	>1600 /s
Simplified network	$\Delta \beta_{W}$	[0, 4]	>2.2

**Fig 1:**
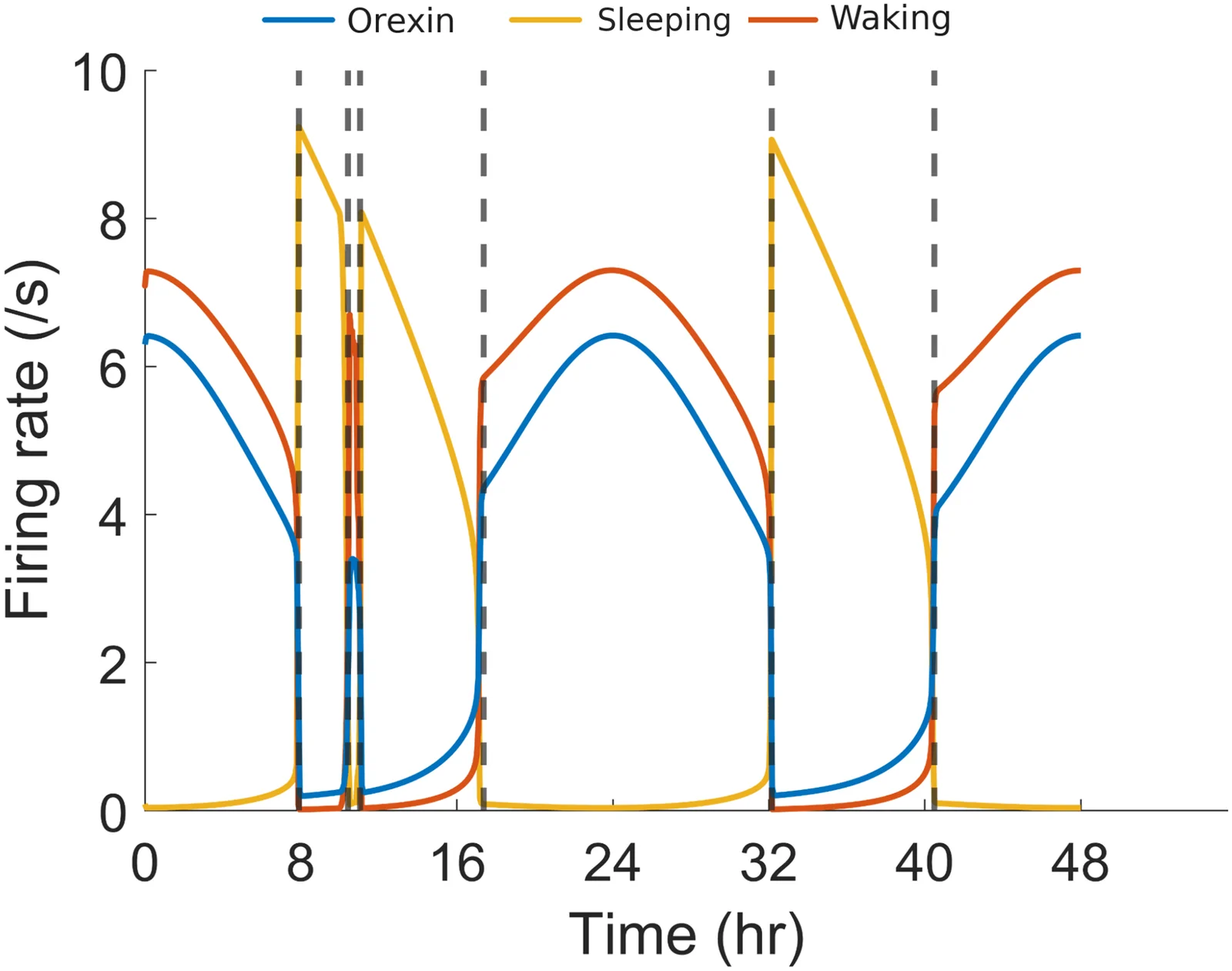
An approximately 30 min nighttime awakening in a 48 hr simulation of the Fulcher et al. sleep model, induced by Equation 29, starting in a waking period. This is presented as an example of a successfully generated awakening with an MA neuronal firing rate comparable to during normal waking periods. In this simulation $\Delta V_{m}=0.01$ mV and $\zeta_{m}(t)$ is sampled from a uniform distribution in the range [0.01,0.02] mV, while all other parameters associated to Equations 14–16 are as in [36] Fig 3. The dashed vertical lines indicate a transition between sleeping and waking periods. This shows nighttime awakenings can be simply induced in the Fulcher et al. model by brute force, but also indicates a high stability in the sleeping state due to its rapid restoration after the perturbative impulse is removed.

Next we implement an identical waking perturbation to the Diniz Behn and Booth model. Equation 29 is added to the LC and DR populations, both associated with waking activity, in Equations 17 and 18. The result of a short perturbation, and a longer and higher amplitude perturbation, is shown in Fig 2 as example simulations of the impact of this added perturbation (NB: [24] was designed to operate on the timescale of the sleep of mice, and hence Fig 2 is over a faster timescale than all other figures in this paper. We have left all parameter values and mechanics of [24] unchanged, other than the introduction of the external perturbation, so that the perturbation’s effects can be isolated). We again investigate the impact of a range of $\Delta V_{m}$’s, this time in the range [0,20000] /s in intervals of 100 /s, with all other parameters fixed as in Fig 2(A). $\Delta V_{m}>1600$ /s is found to be necessary to generate an awakening, defined as a greater LC than VLPO firing rate, which is also summarised in Table 1. This investigation also shows the length of the awakening to be more responsive to the magnitude of $\Delta V_{m}$ than in the Fulcher et al. model, with $\Delta V_{m}=1700$ /s leading to an awakening of 2.5 s, while $\Delta V_{m}=20000$ /s produces one of 266 s. $\Delta V_{m}$’s at and below 1600 /s therefore allow the continuation of the sleeping state, while larger ones lengthen the awakening and further increase the LC firing rate, but not the extent that sleep is not restored after the perturbation. This indicates an otherwise similar situation to the Fulcher et al. model, in that an awakening is readily inducible, but sleep is also restored afterwards, showing sleep to be a potentially highly stable state in these models. The differing amplitudes of $\Delta V_{m}$ required to cause an awakening between this model and the previous are additionally a result of the different units between the models, rather than an informative physiological difference.

**Fig 2:**
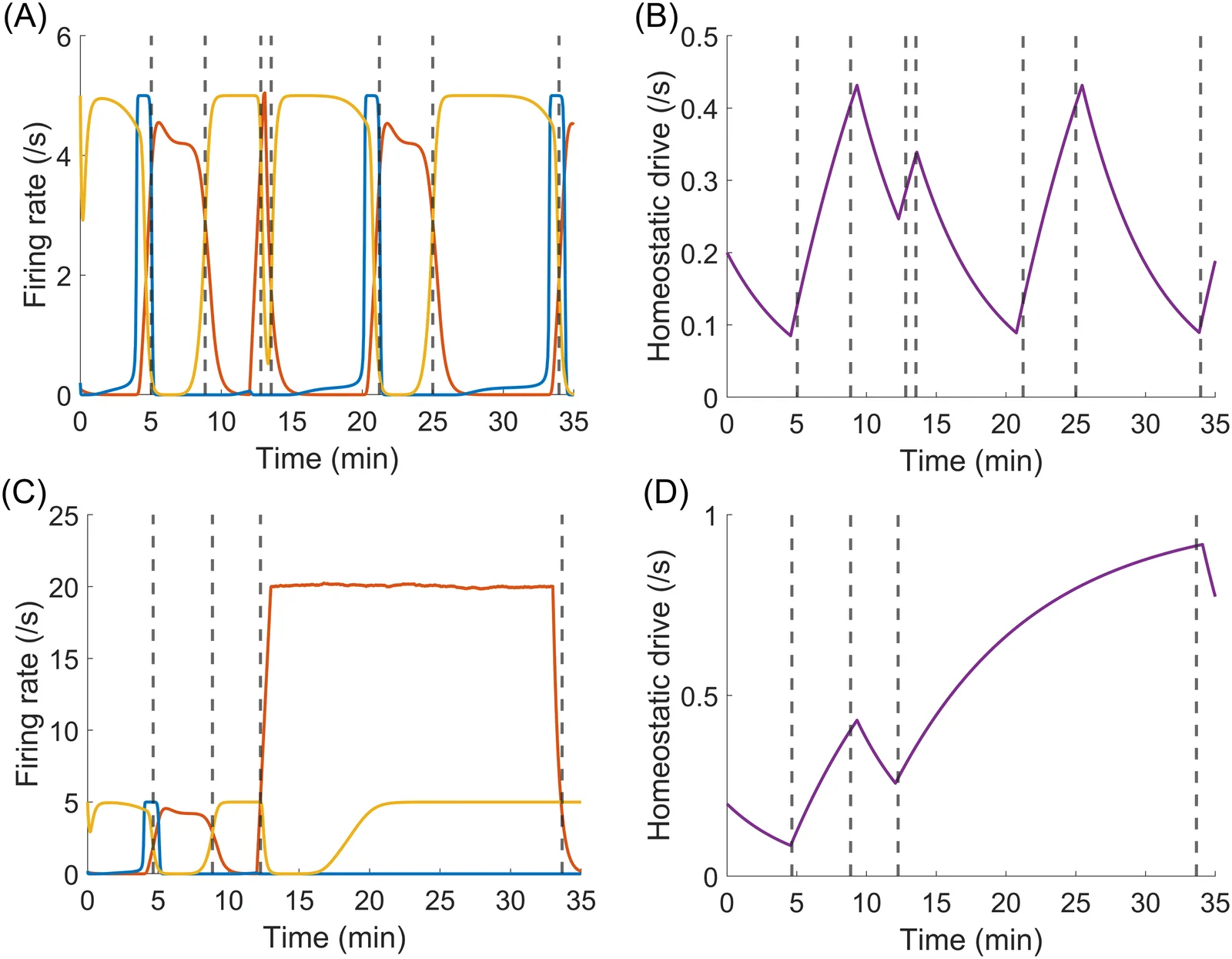
Example nighttime awakenings induced in the Diniz Behn and Booth model, starting in a sleeping period. Unless otherwise stated, all parameters are as in Fig 3 of [24]. (A) the LC firing rate where $\Delta V_{m}=5000$ /s and $\zeta_{m}(t)$ is sampled from a uniform distribution in the range [5000,10000] /s, initiated after 15 minutes lasting for 1 minute, (B) the homeostatic drive in the same case, (C) the LC firing rate where $\Delta V_{m}=20000$ /s and $\zeta_{m}(t)$ is sampled from a uniform distribution in the range [20000,40000] /s, initiated after 12 minutes lasting for 21 minutes, and (D) the homeostatic drive in the same case. This shows nighttime awakenings can also be simply induced in the Diniz Behn and Booth model by a simple perturbation, and that the state once again appears to be stable due to its restoration absent a waking impulse.

We take a similar approach to examining the Diniz Behn and Booth simplified network model [25,67]. To induce nighttime awakenings in this model, we introduce a factor to the threshold parameter of the waking firing rate function, taking a similar form to the previous two perturbations, but adapted in where it is applied to better suit the simplified model’s formalism. This modifies Equation 24 to


$$\begin{array}{c} \frac{dF_{W}}{dt}=\frac{W_{\text{max}}\left(0.5\left(1+\tanh\left(\frac{g_{NW}C_{NX}+g_{RW}C_{RX}+\zeta (t)-\beta_{W}\Delta \beta_{W\infty }(t)}{\alpha_{W}}\right)\right)\right)-F_{W}}{\tau_{W}}, \end{array}$$ (30)

where


$$\begin{array}{c} \Delta \beta_{W\infty }(t)=1+\sigma (t)\Delta \beta_{W}(t), \end{array}$$ (31)

σ(t)∈{0,1} is a switching variable and $\Delta \beta_{W}(t)=\Delta \beta_{W}+\zeta (t)$ is a time-dependent perturbation, with constant $\Delta \beta_{W}$ and noise term ζ(t) sampled from a normal distribution. When an awakening has been triggered and has lasted for less time than the desired duration, σ(t)=1, and 0 otherwise. $\Delta \beta_{W}$ is varied over the range [0,4] in intervals of 0.1, with all parameters fixed and as described in Fig 3(A). A value of $\Delta \beta_{W}>2.2$ is required to produce an awakening, and $\Delta \beta_{W}=2.3$ leads to an awakening of 27 minutes, while $\Delta \beta_{W}=4$ leads to one of 8.3 hours, which is also summarised in Table 1. This is hence more similar to the original Diniz Behn and Booth model, as expected, in that the model is not so stable that significantly higher amplitude perturbations do not produce significantly longer awakenings.

**Fig 3:**
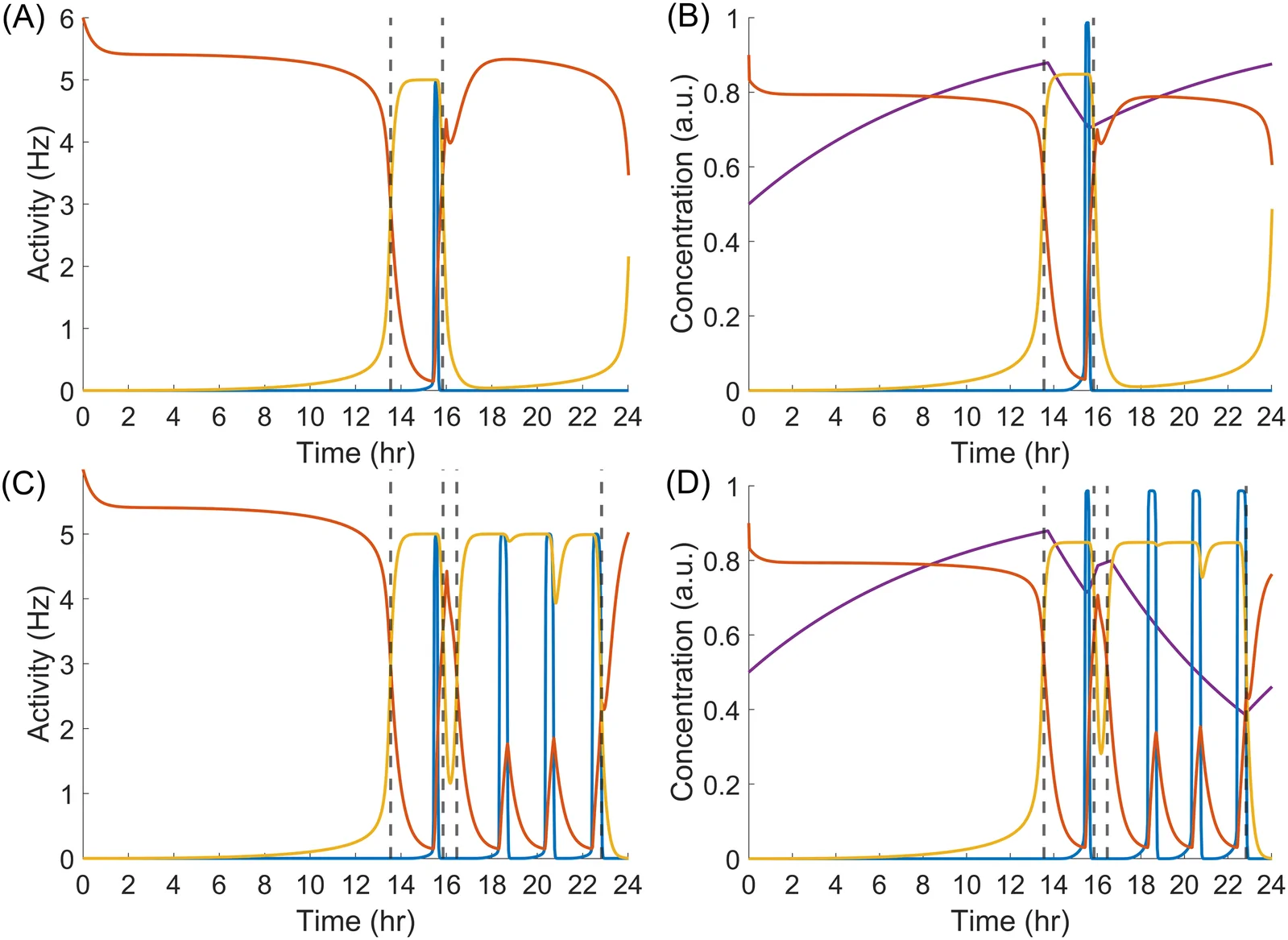
Example nighttime awakenings induced in the Diniz Behn and Booth simplified network model with and without an additional awakening-associated increase to the homeostatic drive, starting in a waking state. The dashed vertical lines indicate a transition between sleeping and waking periods. (A) the activities of the model without the increase, (B) the concentrations without the increase, (C) the activities with the increase and (D) the concentrations with the increase. In (A) and (B) $\Delta B_{W}=3.4$ and ζ(t) is sampled from a normal distribution with mean 0 and standard deviation 0.005. In (C) and (D), $m=1.25\times 10^{-4}$ and c=−5.8 s. In all figures all other parameters are as in Fig 2 of [25]. This shows that while nighttime awakenings can be simply induced in this model as with the two previous, the sleep state is not in general stable and so not restored after the waking impulse is removed. Instead, a modification to increase the homeostatic drive during a nighttime awakening is required to restore sleep for most waking impulse timings through the night.

In contrast to the Fulcher et al. model, the states of the simplified model are in fact not stable. This is demonstrated in Fig 3(A)-3(B), which shows that even after the switch of Equation 30 is disabled, the model continues in the waking state rather than returning to sleep for over 8 hours.

In this model, transitions are primarily driven by the level of the homeostatic drive. Therefore, for the model to be able to restore sleep after an awakening the homeostatic drive must be additionally increased during said awakening. This method is implemented in Fig 3(C)-3(D) with the modification of Equation 31 to


$$\begin{array}{c} \Delta \beta_{W\infty }(t)=1+\frac{\sigma (t)}{\tau_{hs}}(mW+c), \end{array}$$ (32)

where *W* is the time at which the awakening is triggered and *m* and *c* are constants, to Equation 24.

While this method hence allows for control over whether an awakening is short or long, depending on whether the additional homeostatic drive increase is induced, this is still far from an ideal model for an insomnia-related awakening. This is because it requires precise knowledge of the timing and duration of the awakening to calibrate the corresponding effect on the homeostatic drive. If this calibration is not precise, then in addition to the awakening potentially being of an undesired length in either direction, the homeostatic drive may rise to an excessive level, resulting in a breakdown of the model. It is also not clear what the physiological basis for an accelerated increase of the homeostatic drive during awakenings only would be.

In S1 File of the Supporting Information we present further investigations of the stability in the Diniz Behn and Booth and Diniz Behn and Booth simplified network models. This is done by fixing the homeostatic drive at a particular level and then introducing a perturbation during the sleeping state. If once the perturbation ends the system returns to a sleeping state, we consider the state to have been stable, otherwise we consider to be neutrally stable. This process is iterated uniformly over the span of homeostatic drive values indicated in S1 File and Table 2. All parameters other than the homeostatic drive value, including the perturbation magnitudes and timings, are fixed between each simulation. Each simulation is additionally conducted twice, one with the system’s initial firing rate/activity values initialised in a waking state, and once in a sleeping state. If the system transitions from wake/sleep to sleep/wake before any perturbation is added, that initial state is considered to be unstable for that value of the homeostatic drive.

**Table 2:** A summary of the stability of the sleeping and waking states in the Diniz Behn and Booth and Diniz Behn and Booth simplified network models at different homeostatic drive values.

Model	Stability	Homeostatic drive value
Diniz Behn & Booth	Stable wake	(0, 0.23] /*s*
Diniz Behn & Booth	Neutrally stable wake	(0.23, 0.34] /*s*
Diniz Behn & Booth	Unstable wake	≥0.34 /s
Diniz Behn & Booth	Unstable sleep	[0, 0.14) /*s*
Diniz Behn & Booth	Neutrally stable sleep	[0.14, 0.32) /*s*
Diniz Behn & Booth	Stable sleep	>0.32 /*s*
Simplified network	Neutrally stable wake	<0.84
Simplified network	Unstable wake	≥0.84
Simplified network	Neutrally stable sleep	≥0.43
Simplified network	Unstable sleep	<0.43

This is consistent with previous findings that the homeostatic drive is a key component in determining wider model dynamics [67,84]. We summarise the conclusions of these investigations in Table 2, which verify our earlier conclusions as to the high stability of the Diniz Behn and Booth model, contrasted by the significant non-stability of the simplified network model, and were used to inform the perturbation parameter selections in Figs 2 and 3.

We now pause these investigations to review the findings so far against our original hypothesis that simple external perturbations will be sufficient to produce insomnia-like awakenings. We have seen in each of the three models so far that a perturbation can indeed produce an awakening. This is not surprising however, and what is more interesting is the models’ response after the perturbation ends. In the case of the Fulcher et al. model and much of the time in Diniz Behn and Booth model (see Table 2), the end of a perturbation means an assured return to sleep relatively rapidly. In the case of the Diniz Behn and Booth simplified network model and some cases of the original Diniz Behn and Booth model, it means the continuation of a long waking period.

As the ultimate incorporation of both behaviours is necessary for awakenings to be truly insomnia-like, this makes clear that the stability dynamics of these models must be used to allow for this variation. Such dynamics however are not something that could be achieved with simple perturbation of these kind, and so, as expected, further investigation will be required.

We may view the results so far in the framework of dynamical stability analysis, though emphasise that rigorous stability and bifurcation analysis is required for precise identification.

We propose that these results are suggestive of a continuum of stability dynamics – at one end we have Fulcher et al., the sleep state of which appears to be characterised by a wide and deep basin of attraction, and at the other the Diniz Behn and Booth simplified network model, in which the sleep state acts as if a saddle or otherwise with a very narrow and shallow basin of attraction. The Diniz Behn and Booth original model sits in between these points, with a sleep state that is clearly not globally attracting, but with a wider and deeper basin than the simplified network model.

The desirable behaviour for a model of insomnia-like phenomena in this framework is one where the basin of attraction of the sleep state varies within and between nights, narrowing and widening, shallowing and deepening. This would hence produce awakenings that sometimes rapidly decay, and sometimes not decay at all, depending on the characteristics of the basin of attraction at the particular time. A precise identification of these dynamics in these existing models and which parameters and processes control them would indicate whether this can be achieved by parameter modulations, new interactions between existing processes, new processes entirely, or all of these and more modifications besides.

This hence presents two pathways towards models of sleep featuring insomnia-like awakenings that appear equally valid in the first instance. The first, to more comprehensively analyse the dynamical stability of the existing sleep models we have considered to inform major modifications that will allow for the desired forms of variably stable sleeping states. The second, to consider the historical development of these models to this point, incorporate new physiological processes hypothesised to have a role in the functioning of insomnia at these earlier foundational modelling stages, and investigate the development of these new models back into the neuronal population network forms in a similar way to the latter’s original development.

For the remainder of this paper we will focus on the latter approach, and suggest the former as an important methodology to be equally considered in the future.

To this purpose, we now consider the original two process model. This model differs significantly to the three previously considered, which adapted the state switching mechanism into the inhibitory and excitatory interactions of 3 or more neuronal population processes, where waking and sleeping states are categorised by relative amplitudes of the concerned population processes. The two process model, in contrast, includes hardcoded sleep/wake states depending on whether process S last reached the waking or sleeping threshold. In this case rather than add a negative perturbation to reduce process S to the waking threshold and produce an awakening (which would be physiologically analogous to the subject somehow becoming rapidly less fatigued and thus waking up), we add a positive perturbation to the waking threshold, representing an increase in the subject’s propensity to wake. The other dynamic contribution to the sleep/wake thresholds is the circadian drive, which is commonly modelled as a sine wave. To maintain maximum similarity to this existing formalism, we therefore modify Equation 5 to


$$\begin{array}{c} S^{-}(t)=S_{0}^{-}+aC(t)+(b(t)+\zeta (t))\sin\left(\frac{t-\phi }{\delta }\right)\text{ switches to wake.} \end{array}$$ (33)

The addition *b*(*t*) scales the magnitude of an arousal impulse and ζ(t) is a noisy addition to this scaling; both are non-zero only during the perturbation period. ϕ determines the timing of the impulse and δ determines its duration. The sleeping threshold is unchanged. The addition of a perturbation in this way has a less smooth and easily interpretable effect on the model dynamics than in the previous three cases, because the hardcoded switching mechanism means that any perturbation that does not raise the threshold sufficiently to produce an awakening has no lasting impact on the dynamics. In the previous models however memory of any perturbation may be retained, no matter how small, depending on their stability. Whether a perturbation is sufficient to produce an awakening is also far more dependent on the timing of the perturbation than in previous models as a result of this, and so a parameter sweep in *b*(*t*) or ζ(t) to identify a single critical threshold for awakenings would not be meaningful here.

However, in addition to this perturbation to the waking threshold, we find that a uniform increase of the waking threshold constant $S_{0}^{-}$ and decrease to the homeostatic drive sleep decay constant $1/\chi_{s}$ additionally supports arousals of the timings, durations and variabilities that are more consistent with insomnia. We therefore now consider a parameter sweep of the former. $S_{0}^{-}$ is varied over [0.17, 0.4], the lower bound being the value used in [26], in intervals of 0.01 with all other parameters fixed as in Fig 4(A). $S_{0}^{-}>0.3$ was found to be necessary to produce an awakening for these values of *b*(*t*) and ζ(t) at the considered awakening impulse time. However we emphasise again, this exact value will not be the same for different values of *b*(*t*) and ζ(t) or at other awakening times, only that the direc*t*ion of travel, an increase in $H_{0}^{-}$, is physiologically realistic for awakening-based sleep disorders and helps to promote awakenings.

**Fig 4:**
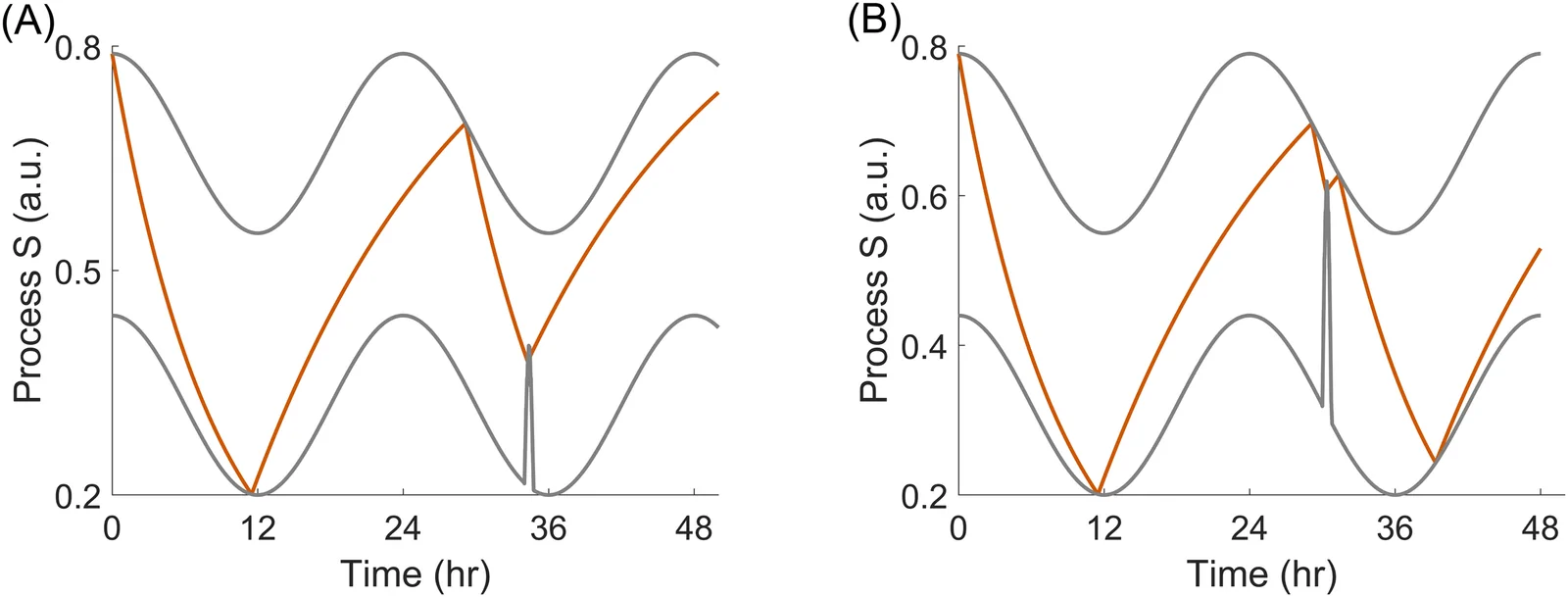
Perturbations to the two process model according to Equation 33. During an arousal impulse *b*(*t*)=0.17 and ζ(t) is sampled from a normal distribution of mean 0.01 and standard deviation 0.005. In (A) ϕ=34 hr and δ=0.75 hr, in (B) ϕ=30 hr and δ=0.75 hr. All parameters are as in Fig 1a) of [26], other than $H_{0}^{-}=0.32$ rather than 0.17 and $\chi_{s}=8.4$ hr rather than 4.2 hr. This shows that once again a nighttime awakening can be induced by a simple added waking impulse, however the behaviour after the impulse is removed is shown to depend significantly on the impulse’s timing.

Decreases to $1/\chi_{s}$ on the other hand do not directly act to cause an awakening, but help to maintain a sleeping rhythm in terms of the timing and length of sleeping periods that is not majorly disrupted, despite the induced awakenings. We therefore do not emphasise any particular value for $1/\chi_{s}$, only that a decrease in the context of insomnia-like awakenings can prevent sleeping periods becoming at highly irregular times or of highly irregular lengths in this model, for example from $\chi_{s}=4.2$ hr to 8.4 hr in Fig 4.

These changes in combination physiologically translate to a general increased bias to the waking state and a less efficient sleeping state, both thought to be factors in many experiences of insomnia [85]. Examples of these perturbations with these two parameter changes are shown in Fig 4.

Thanks to the relative mathematical simplicity of the two process model, these perturbations in fact offer more realistic flexibility to arousal timing and duration than the previous perturbation methods discussed. Rather than being constrained by the dynamical stability of complex interacting sleeping and waking neural populations, the threshold mechanism allows arousals to be triggered at any point in the night. It also physiologically realistically determines their duration based on the simulated subject’s remaining need for sleep. However, while these effects are physiologically realistic in most cases, this model struggles to represent insomnia-like behaviour in which sleep cannot be restored quickly despite a high need for sleep, and is in general too simplified to represent similar disordered behaviour. We nevertheless believe this simplicity forms a strong basis for a new model with the aim of producing insomnia-like awakenings at its core, and now introduce such an original model as a first concrete step towards modelling the complexities and variabilities of insomnia.

### 3.2 A three process approach

This model extends from the same principle of Equation 33, namely transforming the thresholds of the conventional two process model, but this time introduces a comprehensive third process to do so, a wakefulness drive *A*, based in what is known of the physiology of insomnia. This addition of a third process is the most natural extension to the two process model, which maintains the same fundamental framework but extends the number of physiological processes to include those hypothesised as being involved in insomnia-like awakenings. Specifically, drive *A* consists of five components, from left to right in Equations 35–36:

1. A reversion towards an equilibrium, *A*_0_, similar to that of the homeostatic drive.
2. An inverse relation to the homeostatic drive *S*(*t*), ensuring that a higher pressure to sleep produces a lower propensity for wakefulness.
3. (Within the impulse term *I*(*t*), in Equation 36) a physiological wakefulness-increasing impulse with the shape of the difference between rising and decaying exponential functions and triggering at times *t*_0_, representing neurophysiological effects.
4. (Within the impulse term *I*(*t*), in Equation 36) a randomised wakefulness-increasing noisy impulse, representing environmental and psychological effects.

Mathematically, the model is as follows,


$$\begin{array}{cc} \frac{dS}{dt} & =\left(\nu -\chi_{W}S\right)ℋ\left(s\right)-\chi_{S}Sℋ\left(-s\right), \end{array}$$ (34)

$$\begin{array}{cc} \frac{dA}{dt} & =-\frac{A-A_{0}}{\chi_{A}}-k_{S}S(t)+I(t), \end{array}$$ (35)

$$\begin{array}{cc} I(t) & =k_{p}\left(e^{-(t-t_{0})/\chi_{d}}-e^{-(t-t_{0})/\chi_{r}}\right)𝒮(t-t_{0}(r_{t_{0}}))+ℬ(p)𝒩(\mu ,\sigma ), \end{array}$$ (36)

$$\begin{array}{cc} C(t) & =\cos\;\left(\frac{t\pi }{12}\right). \end{array}$$ (37)

Here, *I*(*t*) is hence the combination of physiological and psychological/environmental wake-promoting impulses. This impulse is modified by a step function $𝒮(t-t_{0}(r_{t_{0}}))$ that is 0 when *t* < *t*_0_ (where *t*_0_ is the randomised start time of an impulse) and af*t*er the impulse has decayed to 0, and 1 otherwise. This physiological impulse is modified by a step function $𝒮(t-t_{0}(r_{t_{0}}))$ that is 0 when *t* < *t*_0_ and after the impulse has decayed to 0, and 1 otherwise. For the psychological/environmental impulses, 𝒩 is a set of normal distribution samples and ℬ is a Bernoulli process. ℋ meanwhile is the Heaviside function. The meaning of the parameters, and the values they take in later simulations, is summarised in Table 3. The two processes of the two process model, the homeostatic sleep pressure *S*(*t*) in Equation 34 and the circadian drive *C*(*t*) in Equation 37, are left unchanged from the original model.

**Table 3:** A summary of the physiological meanings and simulations values for Equations 34–39.

Parameter	Value(s)	Physical meaning and value justification
ν	$\frac{5}{91}$	Equilibrium of waking sleep pressure, as in [18,19,26]
$\chi_{W}$	$\frac{5}{91}$	Waking sleep pressure rate constant, as in [18,19,26]
$\chi_{S}$	$\frac{5}{42}$	Sleeping sleep pressure rate constant, as in [18,19,26], and increased for disordered arousals (see main text)
$\chi_{A}$	[0.005, 0.1]	Wakefulness rate constant, ensuring process A operates on neurological hormonal time-scales [88,89]
*A*_0_	0.1	Basal value of the wakefulness drive, comparable to |*C*(*t*)|
$k_{S}$	[0.1, 2]	Influence of sleep pressure on wakefulness
$k_{p}$	[0.1, 2]	Influence of physiological waking impulse on wakefulness
$r_{t_{0}}$	[0.125, 0.5]	Poisson rate of waking impulse, 1–4 expected per night [15]
$\chi_{d}$	[0.005, 0.015]	Waking impulse decay constant, modelled on hormonal response time-scales [88,89] and $>\chi_{r}$
$\chi_{r}$	[0.0015,0.003]	Waking impulse growth constant, modelled on hormonal response time-scales [88,89] and $<\chi_{d}$
*p*	[0.005,0.01]	Bernoulli process probability of psychological and environmental disturbances, ranging from low to high frequency in individual circumstances
μ	[0.01, 0.1]	Normal distribution mean, giving the strength of psychological and environmental disturbances, which vary significantly in individual circumstances [90,91]
σ	[0.01, 0.1]	Normal distribution standard deviation, giving the variation in the strength of psychological and environmental disturbances, which may be significant in individual circumstances [90,91]
$S_{0}^{+}$	0.67	Base sleep threshold as in [18,19,26]
$S_{0}^{-}$	0.32	Base wake threshold, raised for disordered arousals (see main text)
$k_{C}$	0.12	Influence of circadian drive on sleep and wake thresholds as in [18,19,26]
$k_{A}$	[0.1, 2]	Influence of wakefulness on sleep and wake thresholds, ranging from low to high dynamical relevance.

The aforementioned physiological wakefulness-increasing impulse takes the form of a difference of exponentials in order to produce a rapid rise followed by a long decay, consistent with many kinds of physiological impulse responses, including hypothalamic-pituitary-adrenal axis hormonal responses [86], a key function in sleep/wake regulation [87].

The randomised effects of the harder to quantify environmental and psychological components of disordered awakenings such as insomnia are modelled here as the product of a Bernoulli process, ℬ and samples from a normal distribution, 𝒩. As almost nothing is known about the form of physiological responses to psychological or environmental stimulus in the context of sleep disturbances [33], we adopt this stochastic framework to represent their influence without proposing any particular deterministic formulation. Indeed, as both of these external influences naturally will vary hugely between individuals, even regardless of any particular sleep disorder, we do not assert any one stochastic formulation for these influences as more justifiable than any other. Instead we make the choice of a normal distribution as the determinant of the amplitude of the disturbances as it is arguably the most ubiquitous and well-understood statistical distribution, and a Bernoulli process as a similarly mathematically analysable method for controlling the frequency of the disturbances as an intermittent, not continuous, event.

We additionally add a dependence on the wakefulness process *A*(*t*) to both the wake and sleep thresholds,


$$\begin{array}{cc} \text{when }S(t) & =S_{0}^{+}+k_{C}C(t)+k_{A}A(t)\;s=-1, \end{array}$$ (38)

$$\begin{array}{cc} \text{when }S(t) & =S_{0}^{-}+k_{C}C(t)+k_{A}A(t)\;s=1. \end{array}$$ (39)

Thus, not only is there variation in the wake threshold causing the awakenings, but a responsive variation in the sleep threshold, introducing greater variability to the duration of the awakenings.

We first consider a sensitivity analysis of this new model, calculating the total-order Sobol indices of the newly introduced parameters [92], and summary statistics of model awakening outcomes over the randomised simulations used to calculate these indices.

We define 3 outcome categories that are commonly used to characterise sleep quality in the context of awakening disorders such as insomnia [15]. These are total number of awakenings ($N_{A}$) over a 72 hour period (including 3 sleeping periods), the duration of these individual awakenings ($D_{A}$), and timing of these individual awakenings ($T_{A}$) after the beginning of the most recent sleeping period (i.e., if there is an awakening and then a restoration of sleep, $T_{A}$ for a second awakening will be measured from the start of this restoration of sleep). The latter two outcomes are defined in terms of their mean, standard deviation and range of $D_{A}/T_{A}$ for all the awakenings that occur in each 72 hour simulated period, giving a total of 7 outcome measures.

At each selected parameter combination in the Sobol index algorithm, 64 replicate 72 hour simulations are conducted, and the results of the 7 outcome measures are averaged across these 64 replicates. The total-order Sobol indices are then calculated from these outcome measures across the parameter space, indicating how significant each investigated parameter is to determining each outcome measure. The indices for $N_{A}$ and the 3 $D_{A}$ measures are given in Table 4, and for the 3 $T_{A}$ measures in Table 5.

**Table 4:** Total-order Sobol index sensitivity analysis of the three process approach, where an index of 0 implies the parameter has no impact on the corresponding outcome, while 1 implies it maximally determines it, for 4 of the 7 measured outcomes. This shows that parameter $k_{A}$ plays the most significant role in determining $N_{A}$ and $D_{A}$, $k_{S}$ plays a small role in $N_{A}$ but a large one in $D_{A}$, $\chi_{A}$ and $\chi_{d}$ a small role in $D_{A}$, and $r_{t_{0}}$ a small role in $N_{A}$ and the variation of $D_{A}$. All other parameters are shown to be largely insignificant to these outcomes.

Parameter	Influence on $N_{A}$	Influence on mean of $D_{A}$	Influence on standard deviation of $D_{A}$	Influence on range of $D_{A}$
$k_{p}$	0.0027	0.010	0.035	0.019
*p*	0.0028	0.0082	0.036	0.021
μ	0.0037	0.012	0.037	0.023
σ	0.0039	0.013	0.037	0.024
$r_{t_{0}}$	0.13	0.055	0.11	0.088
$\chi_{r}$	0.0089	0.035	0.030	0.085
$\chi_{d}$	0.039	0.14	0.10	0.085
$k_{A}$	0.64	0.83	0.99	0.90
$k_{S}$	0.15	0.48	0.70	0.46
$\chi_{A}$	0.026	0.18	0.22	0.17

**Table 5:** Total-order Sobol index sensitivity analysis of the three process approach, where an index of 0 implies the parameter has no impact on the corresponding outcome, while 1 implies it maximally determines it, for 4 of the 7 measured outcomes. This shows that parameter $k_{A}$ plays the most significant role in determining $T_{A}$, while $\chi_{A}$ and $k_{S}$ play a small role. All other parameters are shown to be largely insignificant to these outcomes.

Parameter	Influence on mean of $T_{A}$	Influence on standard deviation of $T_{A}$	Influence on range of $T_{A}$
$k_{p}$	0.0084	0.015	0.0065
*p*	0.0060	0.015	0.0066
μ	0.0079	0.018	0.0087
σ	0.0095	0.018	0.0077
$r_{t_{0}}$	0.024	0.040	0.0042
$\chi_{r}$	0.016	0.030	0.018
$\chi_{d}$	0.046	0.070	0.057
$k_{A}$	0.95	0.85	0.78
$k_{S}$	0.21	0.14	0.079
$\chi_{A}$	0.096	0.090	0.058

This analysis reveals that $k_{A}$ is overwhelmingly the most significant parameter to determining all 7 outcomes. This is understandable given its role in mediating the impact of almost all additions of this new approach on the value of the sleep/wake thresholds, and therefore on all aspects of triggered awakenings.

$k_{S}$, as the mediator of process S, also plays a significant role in $D_{A}$, with a less but still notable one in $N_{A}$ and $T_{A}$.

Similarly unsurprisingly, the Poisson rate of the occurrence of the physiological waking impulses, $r_{t_{0}}$, has a small but relevant role in $N_{A}$ and the variability of $D_{A}$. This is expected as more frequent awakenings can lead to more of them being of shorter duration, due to a generally higher amplitude of process S as a result of the lack of sleep.

$\chi_{d}$ and $\chi_{A}$, the rate constants of the physiological waking impulses and the wakefulness process, respectively, also have small but not insignificant influences on $D_{A}$, due to their control over how quickly their respective processes reset after an awakening. The latter also has an influence of similar magnitude on $T_{A}$, as it also affects how quickly the wakefulness process, and therefore the sleep thresholds, change to a level where awakenings are more or less easily achieved. All other parameters are shown to be consistently insignificant to the 7 outcomes in comparison to these 5 parameters. This does not, however, mean that they are necessarily irrelevant to other possible outcomes that were not measured, such as the timing and duration of sleep itself.

Given the role of $k_{A}$ as the clearly most significant parameter for awakenings, Fig 5 shows the averaged result (over the 64 replicate simulations at each parameter combination) for each of the 7 outcomes of each parameter combination considered, ordered over the span of $k_{A}$. This helps to show not just that varying $k_{A}$ has an impact on these outcomes, but specifically that increasing it generally increases $N_{A}$ and the variation in $T_{A}$, while decreasing the mean of $T_{A}$. An increasing $k_{A}$ similarly initially increases $D_{A}$ and its variability up to a point, but this increase plateaus earlier in the span of $k_{A}$ than for the $T_{A}$, with even slight reductions in the mean of $D_{A}$ at the top end. This too is largely expected behaviour; with a low value of $k_{A}$ the wakefulness process and thus the waking impulses become insignificant to the model, while a stronger influence allows for more and earlier awakenings, i.e., a lower mean $T_{A}$ but higher variability thereof. As these earlier awakenings are likelier to be shorter than those in the middle of the sleeping period, due to process S being greater earlier in the night, it is expected that more early awakenings would not increase $D_{A}$.

**Fig 5:**
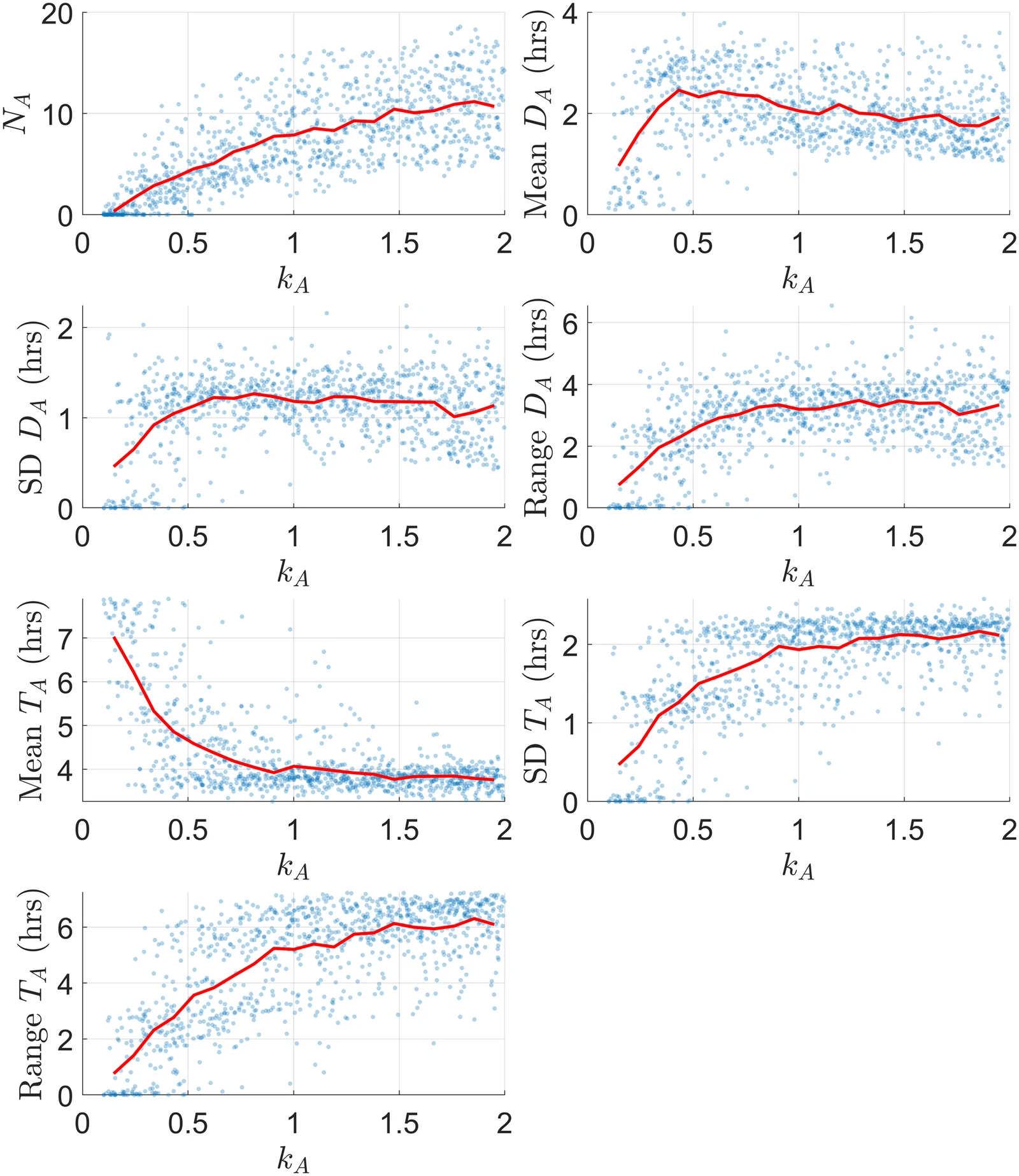
Scatter-plots for each of the 7 outcomes of each simulated parameter combination, ordered over the variation of $k_{A}$. Each blue dot represents a single simulated parameter combination averaged over 64 replicates, while the red line represents the binned mean trend line. This shows that, as predicted by the total-order Sobol indices in Tables 4 and 5, variation in $k_{A}$ has a significant impact on the awakening phenomena produced by the model. Specifically, an increasing $k_{A}$ is shown to increase $N_{A}$, initially increase the mean and variability of $D_{A}$ (but less so after it reaches ~0.5), and increase the variability in $T_{A}$ while reducing its mean.

Similar figures showing how each of the 7 outcomes change as each of the 10 parameters are varied are presented in S1 File of the Supporting Information.

Finally for this analysis, we present the summary statistics of the 7 outcomes across every parameter combination. Fig 6 consists of histogram plots of each 72 hour simulation for each of the 7 outcomes, showing most commonly $N_{A}=4-6$ with a significant frequency across the range 0−16, a mean $D_{A}$ most commonly 1.6−1.8 hours with a significant frequency across the range 1.2−3 hours, and a $T_{A}$ most commonly of 6.5−7 hours with a significant frequency in the range 5−7 hours, 0−0.5 hours and 2−3.5 hours. In the focus of this approach being first and foremost to produce a significant number of awakenings that can vary in number, length, timing and whether or not sleep is restored afterwards, this analysis suggests the approach performs well on these aims. To provide additional context however, we will also now briefly contrast these outcomes to common observations and characterisations of clinical insomnia.

**Fig 6:**
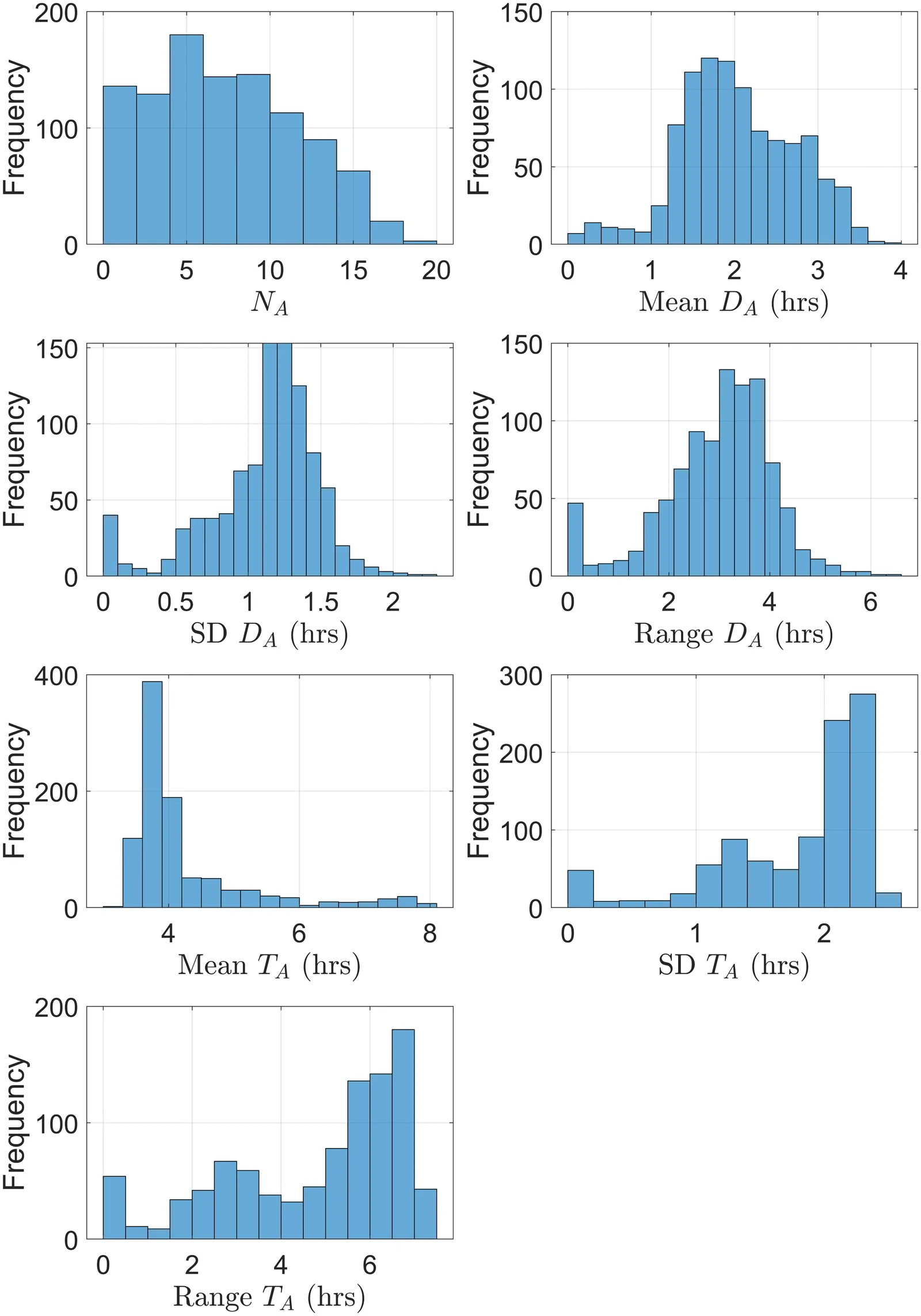
Histograms for the 7 measured model outcomes of each 72 hour simulation across the studied parameter ranges. This shows a high variability in each of the number of awakenings ($N_{A}$), awakening duration ($D_{A}$), and awakening timing ($T_{A}$), demonstrating the potential of the approach to produce a wide range of behaviours. This is an essential feature for application to disorders as variable as insomnia.

As previously discussed, insomnia is an extremely variable disorder consisting of many phenotypes, and there is no definitive way in which any of these phenotypes present across these measurement categories. However, 3 or more awakenings a night for 3 or more nights a week is often considered a threshold for both acute and chronic insomnia [15], which compares favourably to this model most commonly producing up to 4−6 awakenings in a 72 hour period.

Insomnia is even less identified with the duration of individual awakenings, with different, or even the same, patients experiencing individual awakenings lasting from only a few minutes, to a few hours, to never restoring sleep. This model also exhibits a great variability in the duration of awakenings, however individual awakenings of less than an hour may be under-represented from the perspective of insomnia.

The timing of awakenings is similarly highly variable; insomnia is strongly characterised by all of early and late night awakenings, and waking early and being unable to restore sleep at all [16]. This model also demonstrates significant variability in this measure; while the average awakening is strongly clustered in the middle of the night, the high standard deviation and range show this to be because of a significant but similar number of early and late awakenings, rather than because they are exclusively occurring in the middle of the night.

We now present example simulations from the model, to give an intuition of how it functions in comparison to the two process model. Fig 7 shows two simulation iterations of this model with randomised influence of physiological waking impulse on wakefulness, physiological waking impulse decay constant, and timing of physiological waking impulses, using the parameters in Table 6. These simulations exhibit a variety of behaviours, with arousals of both short and long durations, disturbed and undisturbed nights, and sleep prematurely ended by environment and psychological factors. These latter factors can be particularly variable from case to case, and so we additionally present a simulation with much greater influence from these factors in Fig 8, resulting in highly disturbed sleep that cannot be maintained for more than a few hours.

**Table 6:** The parameter values used to simulate Equations 34–39 to produce Figs 7 and 8.

Parameter	Value
ν	$\frac{5}{91}$
$\chi_{W}$	$\frac{5}{91}$
$\chi_{S}$	$\frac{5}{42}$
$\chi_{A}$	0.1
*A*_0_	0.1
$k_{S}$	0.3
$k_{p}$	[1, 1.4]
$r_{t_{0}}$	0.2
$\chi_{d}$	[0.005, 0.015]
$\chi_{r}$	0.002
*p*	0.01
μ	0.1
σ	0.01
$S_{0}^{+}$	0.67
$S_{0}^{-}$	0.32
$k_{C}$	0.12
$k_{A}$	1

**Fig 7:**
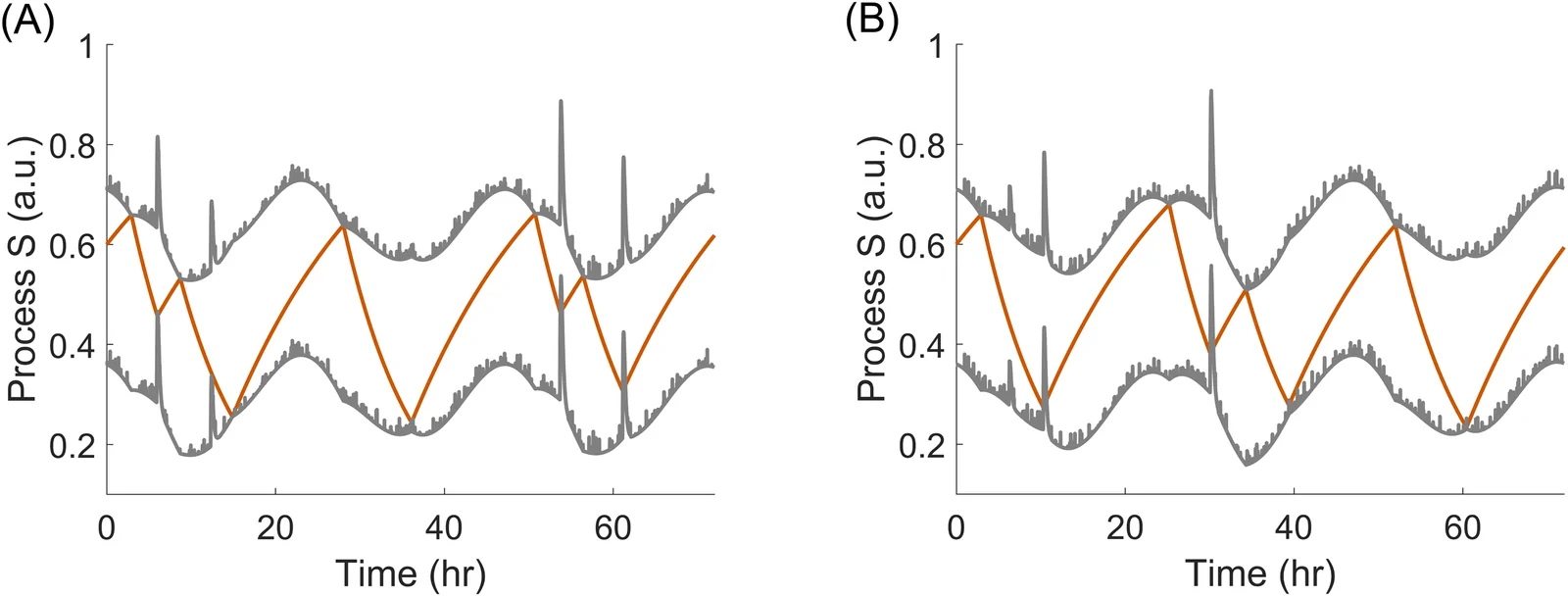
Two example simulations of the original three process model, where the red line is process S given by Equation 34 and the grey thresholds are given by Equations 38–39. Parameters are summarised in Table 6, including the stochastic processes $r_{t_{0}}$, ℬ(p) and 𝒩(μ,σ) and the randomised sampling of $k_{p}$ and $\chi_{d}$, which are re-initialised for each simulation and hence produce a different outcome in (A) and (B), but using the same parameters specified in the table. This shows what an awakening looks like in this model, as well as a night without awakenings, and the scale of the impact of the added physiological impulses relative to the environmental and psychological noise.

**Fig 8:**
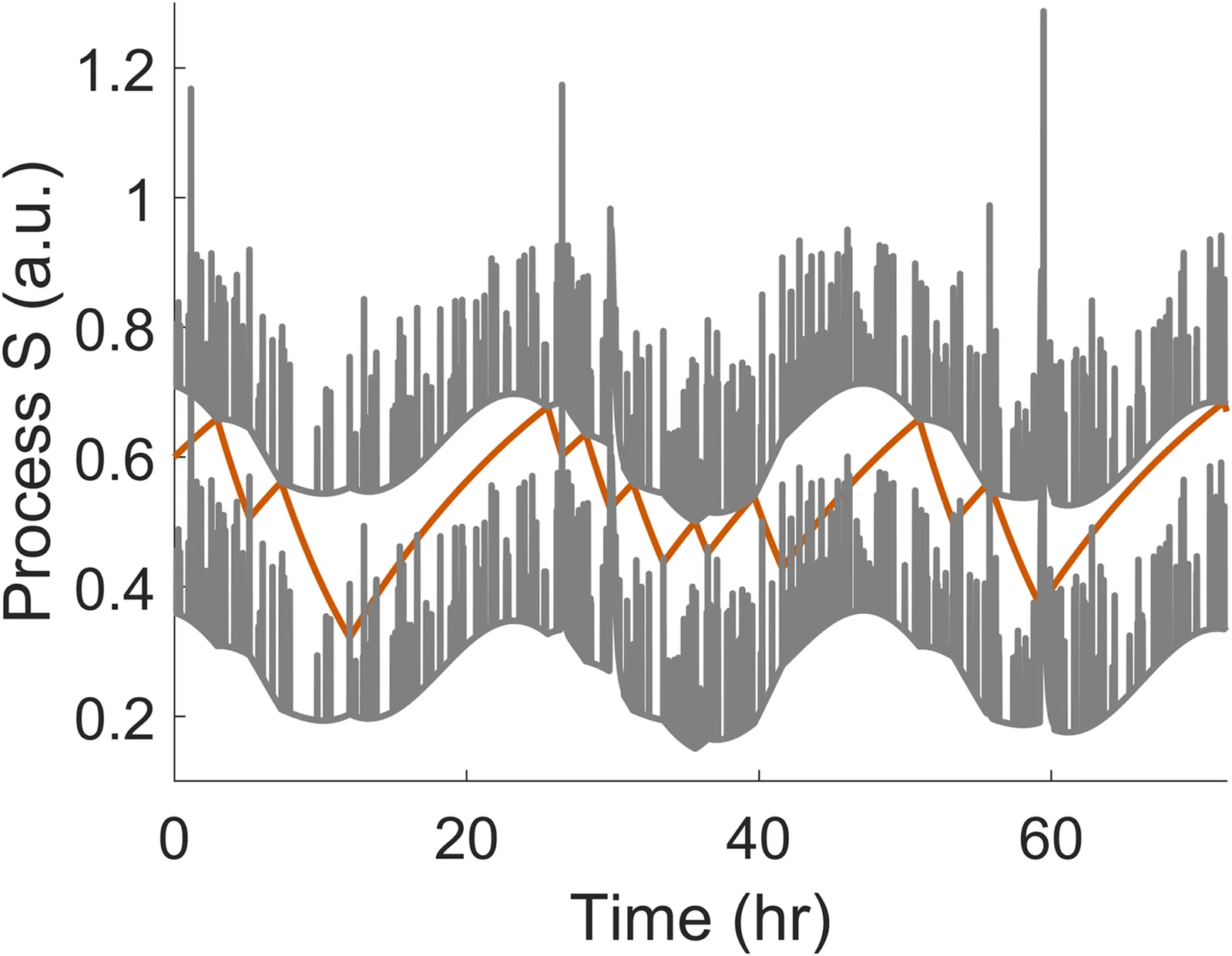
A simulation of the original three process model, where the red line is process S given by Equation 34 and the grey thresholds are given by Equations 38–39. Parameters are summarised in Table 6, with the exception that μ=0.6 rather than 0.1, and σ=0.1 rather than 0.01, to increase the influence of the environmental and psychological wakefulness impulse factors. This shows how the noise can be increased to the level that an undisturbed night’s sleep becomes impossible, and hence how the model can be tuned to apply to circumstances such as extreme environmental noise or psychological distress.

In summary, sensitivity analysis of the wakefulness drive has shown that variation in key parameters allows control over the number, duration and timing of the awakenings generated by the three process model. Further, the wider analysis over every new parameter shows that a majority of the parameter space produces a number of awakenings consistent with the definition of insomnia [15], with the possible variation in their duration and timing that also characterises the disorder. This is therefore a promising approach to mathematical modelling of insomnia phenomena, with much potential for further development, which we discuss in the following section.

### 3.3 Ethics

All data used and produced in this paper are purely synthetic, and therefore ethical approval was not required.

## 4 Discussion

Mathematical modelling of insomnia itself is an extremely complex task, and perhaps cannot even truly be said to be currently possible, whether because of the huge variability in its symptomatic presentations, the lack of entirely objective diagnostic criteria, or because of how little is known of its physiological causes. However, modelling the phenomenon of wakefulness and how awakenings of types recognisable from many presentations of insomnia may provide not only a strong basis for potential future models of ‘true insomnia’, but also advance our present understanding of the physiology of such phenomena when combined with experimental data.

We have approached this aim first through making perturbative additions to existing successful sleep models. Supported by additional analysis of the stability of the processes in contemporary neuronal population models, we find that these complex sleep models are not well suited to simple adaptations to model awakenings due to being either too robustly stable or non-stable. A similar approach to the foundational two process model was more successful, with the simpler threshold sleep/wake switch system better supporting a range of arousal behaviours.

Following these findings, we introduced a new model based on the two process model, making a shift from established models’ primary focus on the processes necessary to reproduce typical sleep, to instead conceive of the processes necessary to produce insomnia-like disordered sleep. This three process model includes a wakefulness quantity, inversely related to the traditional homeostatic drive, and incorporates both randomised physiology-based arousal impulses and environmental and psychological disturbances.

In summary, we have:

- investigated the capacity of existing sleep models to produce insomnia-like awakenings with simple external perturbations, identifying key stability dynamics in these models for controlling insomnia-like awakenings, and proposed further investigation of these dynamics in future work,
- introduced an original mathematical formulation of a third process added to the two process model of sleep, the wakefulness drive, which incorporates insomnia-like nighttime awakening phenomena into the original model,
- analysed the sensitivities of the third process’s original components and the statistical properties of the awakening phenomena it produces, providing a tunable foundation for future development of this approach to model specific insomnia phenotypes and make verifiable quantitative predictions.

This new three drive approach bears similarities to other efforts to build on the two process model, such as the earlier-discussed 2m3d paradigm [33] and inclusion of REM/NREM cycles [27]. The former’s conception of additional drives being required to incorporate a variety of physiological processes beyond the homeostatic and circadian was an inspiration to our development of the wakefulness drive. Similarly, [27]’s method of producing ultradian oscillations contributed to the mathematical form of our physiological waking impulses.

However, neither of these works, nor others, had the aim of including nighttime awakenings into the phenomenology of the two process model. The 2m3d paradigm additionally remained a qualitative one, without proposing specific mathematical forms of a third drive.

We therefore emphasise again the originality of our contribution lying in the mathematical formulation of the wakefulness drive, supporting quantitative evaluation and prediction. Additionally, in taking a three process modelling approach to the problem of nighttime awakenings, we provide a tunable, quantitative foundation for future development towards mathematical models of insomnia.

Just as the original two process model was utilised with real data to advance our understanding of sleep, and spawned more complex and descriptive physiology-based models, so too may this class of three process models focused on wakefulness better our understanding of how and why arousals can occur, and similarly be developed to more complex representations of the neurophysiology involved.

Next steps for this new approach therefore include:

- exploring the feasibility of the same development pathway of the original two process model from the threshold model to a continuous dynamical flip-flop switch for this three process approach,
- investigating its strengths and weaknesses in modelling real data to inform its future development,
- elaboration of the environmental factors considered as part of the third drive based on physiological research and data-based analysis of sleeping patterns, including other factors such as persistent exposure to light at time misaligned to the circadian drive,
- data-driven development of this approach to apply to different phenotypes of insomnia, such as onset insomnia,
- integration of commonly comorbid sleep disorders,
- a comprehensive dynamical stability and bifurcation analysis of the existing neuronal population network sleep models, precisely identifying the attractor landscapes of their sleeping states and their controlling factors. This will identify whether modifications such as including new interactions in the homeostatic drive or whole new processes (as we considered with the three process approach) can modulate these basins so as to produce insomnia-like awakenings.

In conclusion, we have presented two clear, promising pathways for producing insomnia-like awakenings in mathematical models, and eventually models of insomnia phenotypes themselves. This has included original findings on the stability of mainstream sleep models, indicating a framework for how further stability analysis could be applied to incorporate insomnia-like awakenings into these models. Finally, we developed a new three process approach, built on the foundational two process model, that successfully produces awakenings with similar variability to those of insomnia and provides a strong basis for future development of this field.

## Supporting information

S1 Fig The role of the homeostatic drive in triggering wake-sleep transitions in the Diniz Behn and Booth model. The LC firing rate is shown for different values of the homeostatic drive. (A) the LC firing rate for *h* = 0.33 /s, (B) the homeostatic drive for the same case, (C) the LC firing rate for *h* = 0.34 /s, (D) the homeostatic drive for the same case. In (A) and (C) the LC, representing waking, firing rate is in blue, the R, REM, in orange and VLPO, NREM, in yellow.
(TIF)

S2 Fig The role of the homeostatic drive in triggering sleep-wake transitions in the Diniz Behn and Booth model. The LC firing rate is shown for different values of the homeostatic drive. (A) the LC firing rate for *h* = 0.15 /s, (B) the homeostatic drive for the same case, (C) the LC firing rate for *h* = 0.14 /s, (D) the homeostatic drive for the same case. In (A) and (C) the LC, representing waking, firing rate is in blue, the R, REM, in orange and VLPO, NREM, in yellow.
(TIF)

S3 FigThe role of the homeostatic drive in stabilising the sleeping state in the Diniz Behn and Booth model.The LC firing rate is shown for different values of the homeostatic drive with and without waking perturbations. (A) the LC firing rate for *h* = 0.33 /s without a perturbation, (B) the same case with a waking perturbation at 12 minutes lasting for 1 minute, where $\Delta V_{m}=20000$ /s, (C) the LC firing rate for *h* = 0.32 /s, (D) the same case with a waking perturbation at 12 minutes lasting for 1 minute, where $\Delta V_{m}=20000$ /s. The LC, representing waking, firing rate is in blue, the R, REM, in orange and VLPO, NREM, in yellow.(TIF)

S4 FigThe role of the homeostatic drive in stabilising the waking state in the Diniz Behn and Booth model.The LC firing rate is shown for different values of the homeostatic drive with and without sleeping perturbations. (A) the LC firing rate for *h* = 0.22 /s without a perturbation, (B) the same case with a waking perturbation at 12 minutes lasting for 1 minute, where $\Delta V_{m}=-4000$ /s, (C) the LC firing rate for *h* = 0.23 /s, (D) the same case with a waking perturbation at 12 minutes lasting for 1 minute, where $\Delta V_{m}=-4000$ /s. The LC, representing waking, firing rate is in blue, the R, REM, in orange and VLPO, NREM, in yellow.(TIF)

S5 Fig Wake-sleep transitions in the Diniz Behn and Booth simplified network model as influenced by different values of the homeostatic drive. The homeostatic drive, *H*, is kept at a constant level, with the model starting in a waking state. This shows that a value above *H* = 0.83 is required for the model to transition from sleep to wake. (A) the activities of the model with a constant *H* = 0.83, (B) the concentrations of the model with a constant *H* = 0.83, (C) the activities of the model with a constant *H* = 0.84 and (D) the activities of the model with a constant *H* = 0.84. Orange represents the waking population, yellow the NREM, blue the REM and purple the homeostatic drive.
(TIF)

S6 Fig Sleep-wake transitions in the Diniz Behn and Booth simplified network model as influenced by different values of the homeostatic drive. The homeostatic drive, *H*, is allowed to vary according to the original model until reaching the desired level during a sleep phase, at which point it becomes constant. This shows that a value below *H* = 0.43 is required for the model to transition from sleep to wake. (A) the activities of the model with a constant *H* = 0.42, (B) the concentrations of the model with a constant *H* = 0.42, (C) the activities of the model with a constant *H* = 0.43 and (D) the activities of the model with a constant *H* = 0.43. Orange represents the waking population, yellow the NREM, blue the REM and purple the homeostatic drive.
(TIF)

S7 Fig Scatter-plots for the number of awakenings of each simulated parameter combination, ordered over the variation of each of the 10 parameters. Each blue dot represents a single simulated parameter combination, while the red line represents the binned mean trend line.
(TIF)

S8 Fig Scatter-plots for the mean awakening duration of each simulated parameter combination, ordered over the variation of each of the 10 parameters. Each blue dot represents a single simulated parameter combination, while the red line represents the binned mean trend line.
(TIF)

S9 Fig Scatter-plots for the standard deviation of the awakening duration of each simulated parameter combination, ordered over the variation of each of the 10 parameters. Each blue dot represents a single simulated parameter combination, while the red line represents the binned mean trend line.
(TIF)

S10 Fig Scatter-plots for the range of awakening duration of each simulated parameter combination, ordered over the variation of each of the 10 parameters. Each blue dot represents a single simulated parameter combination, while the red line represents the binned mean trend line.
(TIF)

S11 Fig Scatter-plots for the mean timing of each simulated parameter combination, ordered over the variation of each of the 10 parameters. Each blue dot represents a single simulated parameter combination, while the red line represents the binned mean trend line.
(TIF)

S12 Fig Scatter-plots for the standard deviation of the timing of each simulated parameter combination, ordered over the variation of each of the 10 parameters. Each blue dot represents a single simulated parameter combination, while the red line represents the binned mean trend line.
(TIF)

S13 Fig Scatter-plots for the range of the timing of each simulated parameter combination, ordered over the variation of each of the 10 parameters. Each blue dot represents a single simulated parameter combination, while the red line represents the binned mean trend line.
(TIF)

S1 File S1 and S2 Appendices. (PDF)
