# Supplementary material for: A three process approach for insomnia-like awakenings
Source: PLoS One. 2026 Nov 4;21(11):e0360640. doi: 10.1371/journal.pone.0360640 (PMC13734508; doi:10.1371/journal.pone.0360640)
Supplement: Supplementary file 1 [file pone.0360640.s014.pdf]

Supporting information

S1 Appendix

We also consider the impact of different levels of the homeostatic drive on the sleep/wake transitions in the Diniz Behn and Booth and Diniz Behn and Booth simplified network models. In Fig. S1, we show the LC firing rate at different constant values of the homeostatic drive for the former. The homeostatic drive is initially allowed to evolve as per Equation 22, starting from a value of 0.2 /s, until it reaches the desired value, after which it remains constant. This shows that at $h = 0.34$ /s the sleep pressure becomes sufficient to drive a transition from waking to sleeping. Fig. S2 shows the reverse transition, reducing the homeostatic drive from 0.44 /s until reaching a constant value, demonstrating that a homeostatic drive value of approximately 0.14 /s and lower is required to trigger a transition from sleeping to waking.

S1 Fig. The role of the homeostatic drive in triggering wake-sleep transitions in the Diniz Behn and Booth model. The LC firing rate is shown for different values of the homeostatic drive. (A) the LC firing rate for $h = 0.33$ /s , (B) the homeostatic drive for the same case, (C) the LC firing rate for $h = 0.34$ /s, (D) the homeostatic drive for the same case. In (A) and (C) the LC, representing waking, firing rate is in blue, the R, REM, in orange and VLPO, NREM, in yellow.

S2 Fig. The role of the homeostatic drive in triggering sleep-wake transitions in the Diniz Behn and Booth model. The LC firing rate is shown for different values of the homeostatic drive. (A) the LC firing rate for $h = 0.15$ /s , (B) the homeostatic drive for the same case, (C) the LC firing rate for $h = 0.14$ /s, (D) the homeostatic drive for the same case. In (A) and (C) the LC, representing waking, firing rate is in blue, the R, REM, in orange and VLPO, NREM, in yellow.

A combination of these two investigations, perturbations and homeostatic drive levels, reveals further key dynamics of this model. Fig. S3 shows the waking firing rate at two different constant homeostatic drive values, reached in the same way as in Figs. S1 and S2, with and without a waking perturbation. When there is no perturbation it is clear that the change in the homeostatic drive does not itself cause a state transition, and yet at $h = 0.33$ /s the waking perturbation eventually decays back to a sleeping state, while at $h = 0.32$ /s it becomes permanent, initiating a transition from sleeping to waking. This therefore indicates that between these two homeostatic drive values the sleeping state transitions from being stable to neutrally stable, and by extension that the homeostatic drive must play a significant role in the determination of the stability of these model states in general.

S3 Fig. The role of the homeostatic drive in stabilising the sleeping state in the Diniz Behn and Booth model. The LC firing rate is shown for different values of the homeostatic drive with and without waking perturbations. (A) the LC firing rate for $h = 0.33$ /s without a perturbation, (B) the same case with a waking perturbation at 12 minutes lasting for 1 minute, where $\Delta V_m = 20000$ /s, (C) the LC firing rate for $h = 0.32$ /s, (D) the same case with a waking perturbation at 12 minutes lasting for 1 minute, where $\Delta V_m = 20000$ /s. The LC, representing waking, firing rate is in blue, the R, REM, in orange and VLPO, NREM, in yellow.

A similar conclusion can be drawn from examining the stability of the waking state. Fig. S4 shows that a sleeping perturbation decays back to waking at a homeostatic drive value of $h = 0.22$ /s, while initiating a transition from waking to sleeping at $h = 0.23$ /s.

S4 Fig. he role of the homeostatic drive in stabilising the waking state in the Diniz Behn and Booth model. The LC firing rate is shown for different values of the homeostatic drive with and without sleeping perturbations. (A) the LC firing rate for $h = 0.22$ /s without a perturbation, (B) the same case with a waking perturbation at 12 minutes lasting for 1 minute, where $\Delta V_m = -4000$ /s, (C) the LC firing rate for $h = 0.23$ /s, (D) the same case with a waking perturbation at 12 minutes lasting for 1 minute, where $\Delta V_m = -4000$ /s. The LC, representing waking, firing rate is in blue, the R, REM, in orange and VLPO, NREM, in yellow.

Considering the Diniz Behn and Booth simplified network model It is shown in Figure S5 that the homeostatic drive must exceed a value of $H = 0.84$ in order for the model to transition from wake to sleep, while Figure S6 shows that the drive must be below $H = 0.43$ to transition from sleep to wake.

S5 Fig. Wake-sleep transitions in the Diniz Behn and Booth simplified network model as influenced by different values of the homeostatic drive. The homeostatic drive, H, is kept at a constant level, with the model starting in a waking state. This shows that a value above $H = 0.83$ is required for the model to transition from sleep to wake. (A) the activities of the model with a constant $H = 0.83$, (B) the concentrations of the model with a constant $H = 0.83$, (C) the activities of the model with a constant $H = 0.84$ and (D) the activities of the model with a constant $H = 0.84$. Orange represents the waking population, yellow the NREM, blue the REM and purple the homeostatic drive.

S6 Fig. Sleep-wake transitions in the Diniz Behn and Booth simplified network model as influenced by different values of the homeostatic drive. The homeostatic drive, H, is allowed to vary according to the original model until reaching the desired level during a sleep phase, at which point it becomes constant. This shows that a value below $H = 0.43$ is required for the model to transition from sleep to wake. (A) the activities of the model with a constant $H = 0.42$, (B) the concentrations of the model with a constant $H = 0.42$, (C) the activities of the model with a constant $H = 0.43$ and (D) the activities of the model with a constant $H = 0.43$. Orange represents the waking population, yellow the NREM, blue the REM and purple the homeostatic drive.

S2 Appendix

S7 Fig. Scatter-plots for the number of awakenings of each simulated parameter combination, ordered over the variation of each of the 10 parameters. Each blue dot represents a single simulated parameter combination, while the red line represents the binned mean trend line.

S8 Fig. Scatter-plots for the mean awakening duration of each simulated parameter combination, ordered over the variation of each of the 10 parameters. Each blue dot represents a single simulated parameter combination, while the red line represents the binned mean trend line.

S9 Fig. Scatter-plots for the standard deviation of the awakening duration of each simulated parameter combination, ordered over the variation of each of the 10 parameters. Each blue dot represents a single simulated parameter combination, while the red line represents the binned mean trend line.

S10 Fig. Scatter-plots for the range of awakening duration of each simulated parameter combination, ordered over the variation of each of the 10 parameters. Each blue dot represents a single simulated parameter combination, while the red line represents the binned mean trend line.

S11 Fig. Scatter-plots for the mean timing of each simulated parameter combination, ordered over the variation of each of the 10 parameters. Each blue dot represents a single simulated parameter combination, while the red line represents the binned mean trend line.

S12 Fig. Scatter-plots for the standard deviation of the timing of each simulated parameter combination, ordered over the variation of each of the 10 parameters. Each blue dot represents a single simulated parameter combination, while the red line represents the binned mean trend line.

S13 Fig. Scatter-plots for the range of the timing of each simulated parameter combination, ordered over the variation of each of the 10 parameters. Each blue dot represents a single simulated parameter combination, while the red line represents the binned mean trend line.
